# Halogenation in Fungi: What Do We Know and What Remains to Be Discovered?

**DOI:** 10.3390/molecules27103157

**Published:** 2022-05-14

**Authors:** Bastien Cochereau, Laurence Meslet-Cladière, Yves François Pouchus, Olivier Grovel, Catherine Roullier

**Affiliations:** 1 Institut des Substances et Organismes de la Mer, ISOMer, UR 2160, Nantes Université, F-44000 Nantes, France; bastien.cochereau@etu.univ-nantes.fr (B.C.); yves-francois.pouchus@univ-nantes.fr (Y.F.P.); olivier.grovel@univ-nantes.fr (O.G.); 2 Laboratoire Universitaire de Biodiversité et Écologie Microbienne, INRAE, University Brest, F-29280 Plouzané, France; laurence.meslet@univ-brest.fr

**Keywords:** enzymatic halogenation, fungi, halogenases, haloperoxidases, halogenated metabolites, biocatalysis

## Abstract

In nature, living organisms produce a wide variety of specialized metabolites to perform many biological functions. Among these specialized metabolites, some carry halogen atoms on their structure, which can modify their chemical characteristics. Research into this type of molecule has focused on how organisms incorporate these atoms into specialized metabolites. Several families of enzymes have been described gathering metalloenzymes, flavoproteins, or S-adenosyl-L-methionine (SAM) enzymes that can incorporate these atoms into different types of chemical structures. However, even though the first halogenation enzyme was discovered in a fungus, this clade is still lagging behind other clades such as bacteria, where many enzymes have been discovered. This review will therefore focus on all halogenation enzymes that have been described in fungi and their associated metabolites by searching for proteins available in databases, but also by using all the available fungal genomes. In the second part of the review, the chemical diversity of halogenated molecules found in fungi will be discussed. This will allow the highlighting of halogenation mechanisms that are still unknown today, therefore, highlighting potentially new unknown halogenation enzymes.

## 1. Introduction

**Figure 1:**
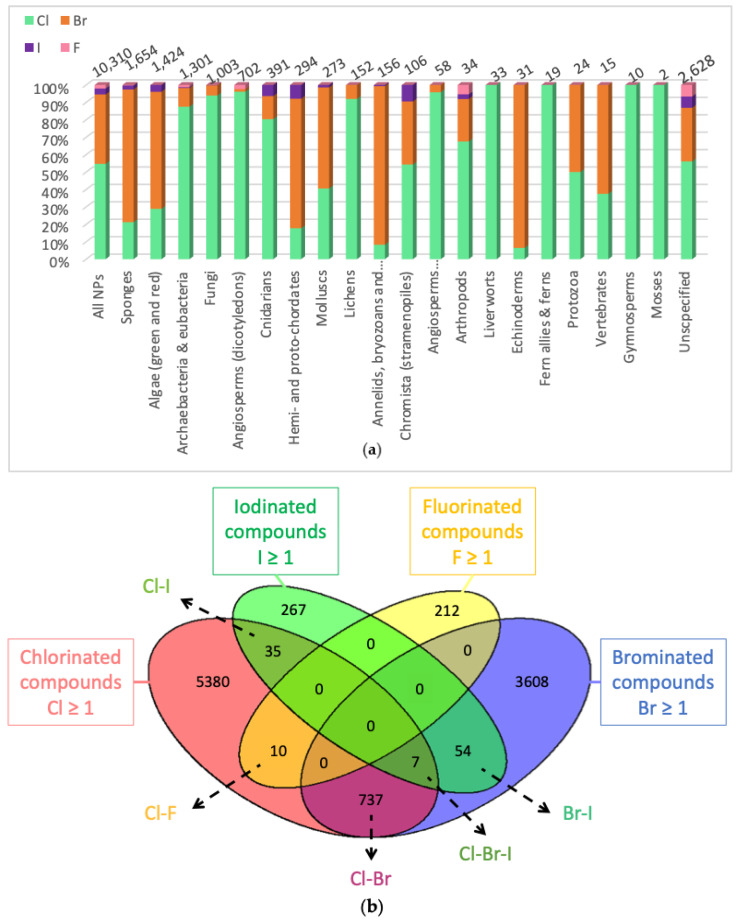
(**a**) Distribution of all halogenated natural products recorded in the *Dictionary of Natural Products* in March 2021 (*n* = 10,310). The compounds were classified according to their clade. NPs in green contain at least one Cl, in orange at least one Br, in purple at least one I, and in pink at least one F. (**b**) Venn diagram of halogen distribution for all the halogenated natural products recorded in the DNP.

While initially considered as extraction artefacts, with only 29 compounds isolated from microorganisms in 1961 [1], halogenated molecules are now well described among natural products. In fact, the first chlorinated compounds were discovered in fungi and lichens at the beginning of the 20th century [2] and the first halogenation enzyme was discovered from a fungus in 1966 [3]. These organisms have pioneered all the studies carried out around halogenation of metabolites in Nature. In March 2021, there were 10,310 halogenated natural products described in the Dictionary of Natural Products (https://dnp.chemnetbase.com/ accessed on 1 March 2021) for all kingdoms of life (with 7725 molecules being associated to a specific type of organism) [4]. This represents 3% of all known natural products. Among them, 59% contained at least one chlorine, 43% contained at least one bromine, 3% contained at least one iodine and only 2% contained at least one fluorine (Figure 1). The most prolific producers of halogenated metabolites are sponges and algae followed by bacteria and fungi, with no distinction between marine or terrestrial strains for these last two clades. Most halogenated compounds contain at least one chlorine but interestingly, brominated compounds are mostly found in marine clades such as sponges, algae, hemichordates, molluscs, annelids and echinoderms (Figure 1). This can be related with that fact that chloride and bromide are 60 and 20 times, respectively, more concentrated in seawater than in sedimentary rocks (SR) found in terrestrial environments ([Cl]: 19,000 mg/kg for seawater against 320 mg/kg for SR, [Br]: 65 mg/kg against 3 mg/kg in SR). Instead, iodide and fluoride are more concentrated in SR than in seawater [5,6], but compounds containing iodine and fluorine are rarer.

**Figure 2:**
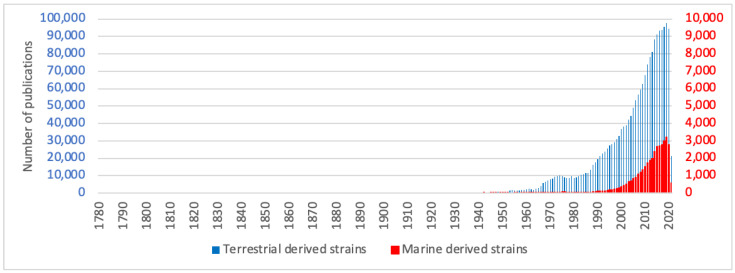
Evolution of the number of publications available on PubMed for terrestrial fungi (blue) and marine fungi (red) over the years (keywords used for PubMed research: (blue) “fungi” NOT “marine”, (red) “fungi” AND “marine”; “fungi” NOT “marine” excludes marine fungi from the research while “fungi” AND “marine” only selects strains from marine environment).

For fungi, chlorinated compounds are the most represented (approximatively 80%). According to The Natural Products Atlas database [7], 705 compounds contain at least one chlorine among the 848 halogenated metabolites described. Only 133 contain at least one bromine, which represents 15% of the described halogenated molecules. However, it should be noted that marine species of fungi have been less studied compared to their terrestrial counterparts. In fact, we now know that fungi are ubiquitous, they are both found in terrestrial and marine environments. They can live on a wide variety of marine habitats including the water column, sediments, and in association to other living organisms like invertebrates, algae or marine mammals. They have been found in many marine areas from the deep environments to the surface water. Many marine strains have their equivalent in the terrestrial environment suggesting strong adaptive capabilities to their living environment [8,9]. Terrestrial fungi were the first species to be studied at the end of the 19th century, with a strong interest after World War two with the discovery of antibiotics such as penicillin. In contrast, regarding the number of publications, studies on marine fungi have only begun at the end of the 1980s (Figure 2). Many more compounds are probably waiting to be discovered as suggested in a recent analysis for halogenated compounds from marine fungi [10].

Nature remains an inestimable source of new bioactive metabolites due to their chemical diversity, their structural complexity and their biological selectivity [11]. From 1981 to 2020, 53% of all new approved drugs were from natural sources (or derived from them). It highlights the importance of natural product (NPs) in the worldwide drug discovery strategies [12], with fungi representing the third kingdom after plants and animals to produce a large diversity of compounds (20,000 new compounds described in 2019). Halogenated secondary metabolites are of interest because halogens have been shown to enhance the bioactivity of their carrier metabolites [13,14]. It should be noted that halogenation can be a critical point for the bioactivity of some natural products, whether in a physiological context in their environment or in the context of biological applications. In the field of drug discovery, the introduction of a carbon-halogen bond can have several effects such as an increase in stability and lipophilicity, in biological membrane permeability and in ligand binding [15]. Indeed, among the drugs that have been released on the market over the last 40 years or among compounds in clinical trials, more than 30% bear one or more halogens in their structure [12,16]. Concerning the roles of halogenated compounds in the marine environment, studies have suggested their anti-biofouling and anti-microbial activities [17,18].

**Figure 3:**
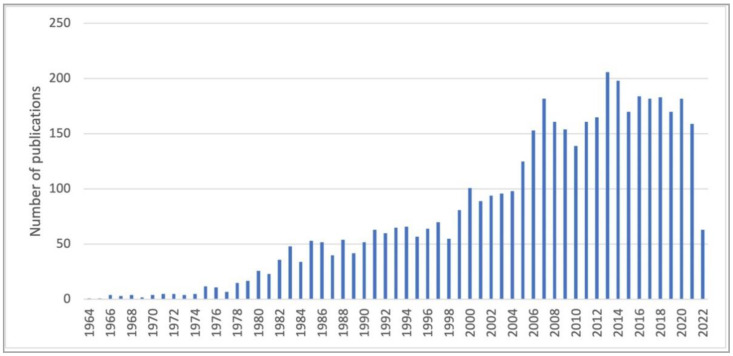
Evolution of the number of publications available on PubMed for halogenation enzymes (keywords used for the research on PubMed: haloperoxidase, chloroperoxidase, bromoperoxidase, iodoperoxidase, and halogenase).

To produce halogenated metabolites, living organisms possess specific complex enzymatic machineries. Enzymes performing halogenation have been widely studied since the 1960s and the discovery of the first haloperoxidase from the fungus *Caldariomyces fumago* [3]. Halogenation enzymes are now divided in different classes depending on the chemical mechanisms used for halogen incorporation in metabolites. So far, halogenation enzymes described include: (1) haloperoxidases and flavin-dependent halogenases, which catalyse electrophilic incorporation of halides; (2) non-heme iron-dependent halogenases, which catalyse free-radical attack of halogen on specific substrates and (3) S-adenosylmethionine-dependent halogenases, which catalyse nucleophilic substitution on aliphatic chains. Regarding the potential of halogenated compounds, especially for drug discovery programs but also for synthetic chemistry applications of all kinds, an increasing interest for these enzymes has grown (Figure 3).

In fact, the development of useful methods for the regioselective halogenation for the construction of functional materials has engaged chemists for many decades [19,20]. The main reagents used for this purpose present different drawbacks such as being irritating, toxic, corrosive and difficult to handle (ex: X_2_ with X = Cl, Br, I) or lacking regioselectivity (ex: *N*-halosuccinimides). In this context, halogenation enzymes represent interesting alternatives towards safer, greener and more efficient chemistry. 

As fungi represent a sustainable source of both compounds and enzymes with a large variety of metabolites produced, this review intends to focus on the different enzymatic classes of halogenation enzymes and the different halogenated metabolites which have been detected or described in fungi until today. The whole fungal kingdom will be investigated including both marine and terrestrial species, even if the marine environment seems to be more favourable to the production of halogenated metabolites [5,21,22]. For each enzyme class, its historical discovery will be developed, and the current knowledge level will be specified. The number of sequences detected and their natural occurrence in fungi will be analysed. Each enzymatic mechanism will be precisely described, and the different three-dimensional structures will be compared (when possible). Associated metabolites will be used as examples when their biosynthetic pathways have been clearly elucidated and involve a specific halogenation enzyme. On the other hand, utilization in biocatalysis will also be discussed and examples of biotransformation will be presented.

## 2. Halogenation Enzymes

### 2.1. Methodology 

Gathering of available data about halogenation enzymes in fungi was done using the Universal Protein Resource (UniProt) database. Research on UniProt was carried out in April 2021 with keywords and restraining the queries in the fungal kingdom (“heme haloperoxidase”, “flavin-dependent halogenase”, “vanadium haloperoxidase” and “non-heme halogenase”) (Table 1). UniProt database included 225,013,025 protein sequences from all kingdoms of life, with 565,928 sequences manually annotated and characterized from experimental results. The rest of the sequences corresponds to high quality computationally analysed sequences with automatic annotation and classification. For this study, Blastp analysis was also performed on the MycoCosm database from Joint Genome Institute which included 2172 fungal genomes available for comparative genomics analysis in February 2022 [23,24]. According to Blastp, the expected value (E) is a parameter that describes the number of hits one can “expect” to see by chance when searching a database of a particular size. E value describes the random background noise and the closer to zero, the more significant the match is. Hits found on MycoCosm database are linked with a predictive Protein family code (Pfam) which are available on the InterPro database. Protein Data Bank (PDB) has been used to obtain the 3D structure of crystallized proteins. To carry out sequence alignments, the software Mega X was used [25]. The PyMOL Molecular Graphics System, Version 2.0 Schrödinger, LLC was used to represent 3D structures. On the PDB, structures were viewed and illustrated using Mol* [26] and RCSB PDB viewer.

### 2.2. General Considerations about Fungal Halogenation Enzymes

#### 2.2.1. Detection and Characterization of Halogenation Enzymes

During the 80s, to detect gene encoding halogenation enzyme in fungal strains, PCR methods were used. For example, to obtain the *C. fumago* CPO mRNA, a specific oligonucleotide probe called “29mer” was designed. This primer targeted chloroperoxidase amino acid residues 167 to 177 which correspond to the signal peptide which is cleaved during secretion of the protein in the culture medium [43]. PCR screening can be used today if the fungal genome is not sequenced. Nowadays, computational methods are the easiest way to detect potential halogenating proteins for example using genome-mining tools like antiSMASH or Blastp [24,44]. These genome-mining methods are very useful for the detection of enzymes present in biosynthetic gene clusters (mainly for FDHs) [45,46,47]. 

Following the discovery of CPO in *C. fumago*, Morris and Hager developed a spectrophotometric assay using monochlorodimedone (or MCD) (Figure S1), which structure closely resembles to 2-chlorocyclopentane-1,3-dione (**1**), the precursor of caldariomycin [48]. In this assay, the formation of dichlorodimedone (or brominated MCD) by the enzyme is spectrophotometrically followed at 290 nm (in the presence of MCD, halide, H_2_O_2_ and vanadate) [48]. This assay is used today for haloperoxidases to determine halogen specificity for chlorine and bromine. For iodine, the thymol-blue (thymolsulfonphtalein) assay can be used to characterize specificity as well as the *o*-dianisidine assay in native protein gel directly [49,50]. Because of the higher specificity of FDHs, enzymatic characterization of this family of enzyme has to be done with the specific substrate of each protein [30,47].

#### 2.2.2. Production of Halogenation Enzymes

Detecting an enzyme constitutes the first step, but to be characterized and used in biocatalysis, enzymes need to be purified. This is one of the most difficult part of the research. It can be very time-consuming, and many troubles can cause inactive proteins, unexpressed proteins, low yield or impure proteins [51]. Trying to produce fungal eucaryotic protein in procaryotic systems can also be an important lock to lift because of the lack of post-translational modification in procaryotic systems which can cause protein aggregation or inactivation [52]. While several eucaryotic expression systems exist, *E. coli* remains the simplest to use [51]. To overcome this technological barrier, chaperones protein can be used to enhance refolding of heterologous enzymes [53]. In the present manuscript, some enzymes presented have been heterologously expressed for biocatalysis experiments like the CPO from *C. fumago*, the vCPO of *C. inaequalis* or the Rdc2 enzyme from *M. chlamydosporia* as well as the MalA protein from *M. aurantiaca* (see Table 2 and Table 3 for produced metabolites and Section 2.3.6, Section 2.4.6 and Section 2.6.5) [30,54,55,56]. Nevertheless, while more complicated and time-consuming, the way to obtain the best activity for purified enzymes remains to natively purify them from the fungal strain as performed for CPO from *C. fumago* and vCPO from *C. inaequalis* [57,58].

### 2.3. Heme-Dependent Haloperoxidases

#### 2.3.1. History

This halogenation enzyme class was the first class to be discovered in fungi during the 60s with a first extracellular heme-dependent chloroperoxidase (CPO) described from *Caldariomyces fumago* [48]. Isolation of CPO by Hager et al. followed their studies on caldariomycin biosynthesis [3]. Heme-dependent haloperoxidases (hHPOs) use a ferriprotoporphyrin IX as prosthetic group and are dependent on hydrogen peroxide. Haloperoxidase enzyme class is characterized by the use of hydrogen peroxide as chemical oxidant [72].

#### 2.3.2. General Structure

**Figure 4:**
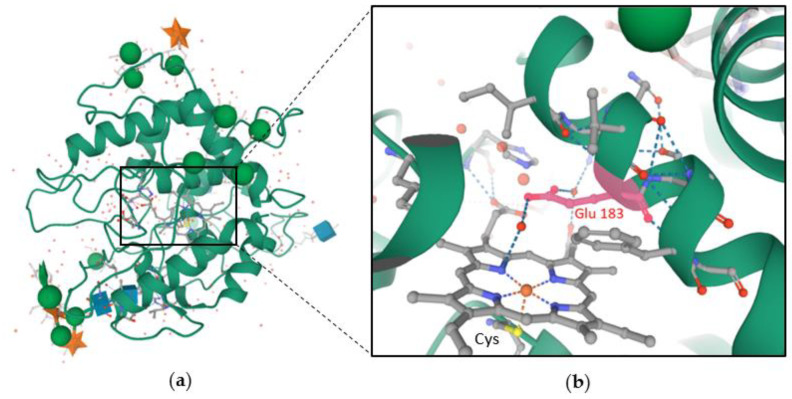
(**a**) Crystal structure of heme-chloroperoxidase from *Leptoxyphium fumago* (PDB ID: 1CPO). (**b**) Active site with glutamic acid 183 colored in pink.

**Figure 5:**
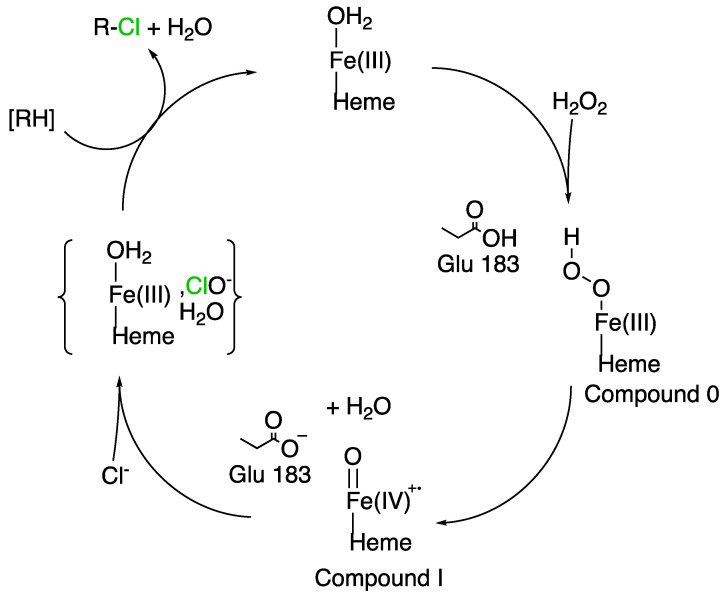
Schematic mechanism of hHPOs inspired by [75].

In the PDB, there is only one fungal heme-dependent haloperoxidase protein which has been structurally resolved. This is the protein which was found in *C. fumago* [73]. The specific gene encodes a protein of 373 amino acid residues, but the mature protein only contains 299 amino acids. This difference was explained by the two cleavages occurring when the protein is excreted (one for the signal peptide at the N-terminus and one at the C-terminus). This enzyme undergoes at least two post-translational modifications, glycosylation and cyclisation [27]. CPO from *C. fumago* was described to be heavily glycosylated, 25–30% of the protein is carbohydrate, glucosamine and arabinose are the two major constituents [3]. Production of recombinant CPO in *Escherichia Coli* has shown that glycosylation is not a mandatory requirement for chloroperoxidase refolding and non-native enzyme activity [74]. As well, over- glycosylation does not affect protein activity too [55]. Inside CPO, heme is stabilized by hydrophobic and hydrogen-bonding interactions in a specific pocket (Figure 4). A proximal glutamic acid participates to the enzymatic mechanism by assisting in the formation of compound 0 and compound I (Figure 5) [75]. In absence of halide as substrate, CPO function is modified and is closer to cytochrome P450 function. Up to now, apart from the CPO from *C. fumago,* there is no other fungal hHPO structure available for comparison [63]. Because of their poor stereoselectivity, identification of related metabolites in fungal metabolome is complex.

Structural analysis of 1CPO (Figure 4) shows similarities between hHPOs and other heme enzymes but heme-dependent haloperoxidases folds into a specific tertiary structure characterized by eight helical segments [73]. Structure of hHPO is unique but as previously said it shares features with both peroxidases and cytochrome P450. The heme cofactor anchorage residue is, like cytochrome P450, a cysteine (Cys 29, Figure 4b) and the distal side of heme is polar, like peroxidases. But the particularity of hHPO is the substitution of the histidine found in peroxidases by a highly conserved glutamic acid as the catalytic acid base [76].

#### 2.3.3. Enzymatic Mechanism and Specificity

The catalytic cycle of the enzyme (Figure 5) begins with the attack of the iron (III)-water complex by hydrogen peroxide which displaces the water molecule. The hydrogen peroxide reacts with a close glutamic acid residue (Glu183 in 1CPO, Figure 4b) to be deprotonated. An iron (III)-hydroperoxo complex is formed. A radical species is then formed with the protonation on the terminal hydroxyl group (which can be hypothesized to come from the Glu183). The radical species ferryl-(IV)-oxo heme cation is named “compound I” which is a versatile oxidative species able to react with a lot of substrates at neutral pH (aliphatic substrates, olefins, sulphides and others presented in Table 2). Here, compound I reacts with halides (X^−^, except F^−^) to form an iron (III)-hypohalide complex at low pH [75]. Structural and crystallographic analysis suggest that a hypohalous acid species is released into the proximal environment because no binding pocket for substrates has been described in hHPOs. The electrophilic XO^−^ (X^+^ donor) then normally reacts with substrates close to the protein [72]. Consequently, regioselectivity and stereoselectivity of hHPOs is quite poor [77]. hHPOs can react with chloride, bromide and iodide but not with fluoride and are named after the high electronegative halide they can oxidize. The formation of compound I and its reaction with halide is very fast. The bounded structure should exist in a rapid equilibrium before expulsion of the hypohalous acid from the heme centre [78]. Because of the low specificity for substrates, identification of the halogenation targets is more difficult. If no substrate is present in the enzyme environment, hypohalous acid reacts with a second equivalent of hydrogen peroxide to produce dioxygen and halide. 

For many years, research teams have debated the fact that there were two halogenation routes for secondary metabolites. The first one was that the hypohalous acid remained inside the hHPO to halogenate the substrate directly after its formation. On the other hand, other researchers claimed that this high reactive species diffuses to the proximal environment to react with acceptable substrates. Today, the second option is favoured and most probably true. A study based on the analysis of 35 chlorinated compounds with different reactivities and sizes proved that the hypohalous acid produced by the hHPO adopts the same behaviour as a hypohalous acid chemically produced. This indicates that the chlorine transfer to the final substrate occurs most probably outside the hHPO by diffusion [79].

#### 2.3.4. hHPOs in Fungi

To evaluate the hHPOs presence in fungi, research on UniProt database has been made using the filters: heme and haloperoxidase and fungi. There were 5088 hits in fungal organisms but only 6 sequences had been studied with the protein function being biochemically characterized in 2022. The other sequences remain uncharacterized and have only been computationally detected in fungal genomes. Only one out of the six characterized proteins has been studied for its halogenation capacities, the CPO from *C. fumago* (Table 1). Among the 6 studied proteins, were the aromatic peroxygenases AaP from *Agrocybe aegerita* and the lignin peroxidase LiP from *Phanerochaete chrysosporium*. These enzymes have been described to possess a secondary haloperoxidase activity, after positive reaction to the monochlorodimedone (MCD) assay, explained hereafter [80,81]. Indeed, the AaP peroxygenase can use bromine to halogenate MCD during the MCD assay. It shares 21% identity with CPO from *C. fumago,* and a crystal structure is available for this enzyme (PDB ID: 2YOR). Piontek et al. who described this activity for AaP thought it was physiologically questionable because of the rarity of bromine in the terrestrial environment [82].

In a second approach, a Blastp analysis was performed using the CPO sequence from *C. fumago* on MycoCosm. With an E-value threshold of 1.0 × 10^−25^, only 6 hits were found. The percentage of identity ranged from 38% to 57%. Interestingly, when the same research was carried out with an E-value threshold of 1.0 × 10^−10^, there were 2360 hits. This high number of sequences is explained because of the multiple presence of genes encoding heme-dependent haloperoxidase in one fungal genome [83]. The general feature of all these hits is that they possess the more or less conserved heme-binding pocket which class them into the peroxidase family 2 (Protein family code Pfam: PF01328). This enzyme family gathers the particular peroxidases, with low similarity with other peroxidases. This family is represented by the hCPO of *C. fumago* and the aromatic peroxygenase from *A. aegerita*. 

#### 2.3.5. Implication in Biosynthesis of Halogenated Compounds

**Figure 6:**
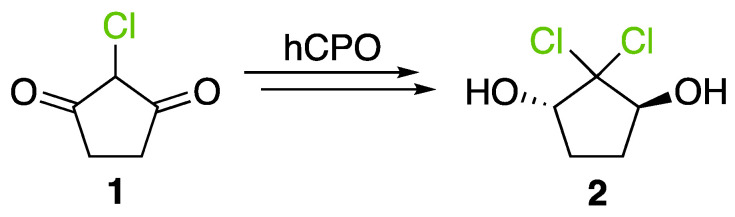
2-chlorocyclopentane-1,3-dione (**1**) chlorination to form caldariomycin (**2**) inspired by [84].

hHPOs have a lot of functions like cytochrome P450 or peroxidase but hHPOs are also thought to be responsible for halogenation of metabolites in fungal metabolome. However, so far, apart from caldariomycin (**2**), no halogenated compound has been related to a hHPO (Figure 6). It is difficult to associate the correct substrate with the corresponding hHPOs because of the external reaction of halogenation and because hHPO encoding genes are found directly in fungal genomes outside biosynthetic gene clusters (BGC), with multiple copies.

#### 2.3.6. Applications and Prospect

CPO from *C. fumago* has been intensively used by fine chemical industries with purified recombinant protein or purified native protein [55,58]. For example, it has been used for its halogenating activity but also for its other activities like oxygen transfer reactions, asymmetric epoxidations of olefins, allylic, benzylic and propargylic hydroxylations, asymmetric sulfoxidations and regioselective oxidations of indoles [59]. The problem with hHPOs is that they are readily inactivated by the oxidant which causes the irreversible oxidative destruction of the porphyrin ring [85]. To improve stability of hHPOs, some research teams have used site-directed mutagenesis to generate mutants with better thermal and oxidative stability [86]. A comprehensive review from Höfler et al. gathers the many chemical applications using CPO from *C. fumago* and AaP peroxygenase from *A. aegerita.* However, some issues still need to be improved to render haloperoxidases more practical in use for large-scale organic synthesis (substrate loadings, selectivity, stability and others enzymatic parameters) [63] (Table 2). 

### 2.4. Vanadium-Dependent Haloperoxidases

#### 2.4.1. History

Vanadium-dependent haloperoxidases (vHPOs) were first discovered during the 80s from the brown algae *Ascophyllum nodosum* [87]. Like heme-dependent haloperoxidases, vHPOs catalyse electrophilic halogenation using the diffusion of hypohalite species (HOX equivalent to “X^+^”). The first vHPO described in fungi was from the terrestrial fungus *Curvularia inaequalis* in 1995 [28].

Like hHPOs, vHPOs are metalloenzymes which stabilise a metallic prosthetic group in their structure. For vHPOs, it is the vanadium oxyanion vanadate (VO_4_^3−^) with an oxidation state of +5 [88]. Vanadium-dependent haloperoxidases can be found in three different forms: vanadium chloroperoxidases (vCPOs) which can use chloride, bromide and iodide, vanadium bromoperoxidases (vBPOs) which can use bromide and iodide and vanadium iodoperoxidases (vIPOs) which only use iodide. vHPOs are named after the most electronegative halide they can oxidize. No vanadium fluoroperoxidases have been discovered yet. It can be explained by the overall thermodynamic potential of hydrogen peroxide which is too weak to oxidize fluoride [75]. It is important to note that vanadium, which is the second most abundant transitions metal in seawater, is abundant in marine environment [89].

#### 2.4.2. Structure

The only crystal structure of a fungal vHPO is from *C. inaequalis* (Table 1). As said previously, vHPOs stabilize a required vanadate prosthetic group. vHPOs share a relatively low level of overall protein sequence identity between the different taxa (algae, fungi, marine bacteria). Only two short domains in the vanadate binding pocket are highly conserved [90], as presented hereafter.

(1) PxYxSGHA
(2) LxxxxAxxRxxxGxHxxxD

**Figure 7:**
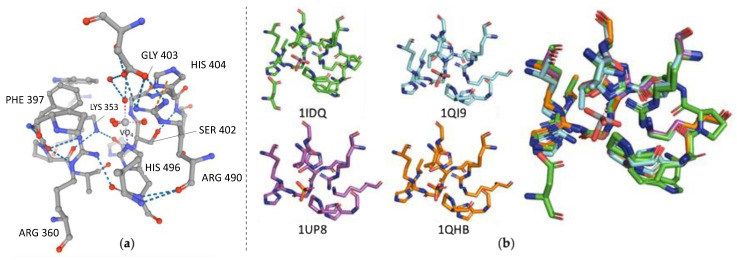
(**a**) Vanadate (VO_4_^3−^)-binding pocket in vCPO from *Curvularia inaequalis* (PDB ID: 1IDQ). (**b**) Structural overlay of active sites from different vHPOs crystallized: vCPO from *C. inaequalis* (PDB ID: 1IDQ), vHPO from *A. nodosum* (PDB ID: 1QI9), vBPO from *C. pilulifera* (PDB ID: 1UP8), and vBPO from *C. officinalis* (PDB ID: 1QHB).

Inside this catalytic pocket, vanadate is coordinated in a trigonal bipyramidal and forms a metallic interaction with the imidazole chain of the highly conserved histidine residue (H496 in *Ci*vCPO) (Figure 7a). In the Protein Data Bank, vCPO 1VNC (and its mutants 1VNE, F, G and H) is the first crystal structure in 1996 and 1999 for its mutants [54,91]. Another structure, the 1IDQ, represents the crystal structure of native vanadium-containing chloroperoxidase from *C. inaequalis* without the azide molecule present in 1VNC [57].

Oxygens in the equatorial plan of vanadate are stabilized by multiple hydrogen bonds with conserved residues (for vCPO PDB ID: 1VNC or 1IDQ-Lys353, Arg360, Ser402, Gly403 and Arg490) [88,92].

Figure 7b shows the active site highly conserved in vHPO from different taxa (algae and fungi here). There is a perfect overlay of the residues which stabilize the vanadate cofactor [90]. In the vCPO from *C. inaequalis*, another histidine residue is proposed to be part of the catalytic cycle (H404). This same histidine residue is observed in the vBPO from *A. nodosum*. The intermediate peroxovanadate formed during catalysis is oriented toward Phe397. In fungi, there is one equivalent of vanadium per enzyme. In contrast with vHPOs from algae which revealed multimerization, vCPO from *C. inaequalis* is composed by only one monomer. In fungal vHPOs, the active site neighbours the N-terminal helix bundle [90]. Multimerization seems to be a common feature in algal vHPOs producing high molecular mass protein but, as shown in Figure 7, the active sites of each monomeric form can be overlaid. Nonetheless, monomeric algal subunits and vCPO from *C. inaequalis* have a different tertiary structure. Algal subunits contain more β-structures than vCPO. Comparing vCPO from *C. inaequalis* and the homodimeric vBPO (I) from *A. nodosum*, the enzyme core is similar: vCPO contains a two α-helix bundles while in vBPO, each monomer provides one bundle to form the two α-helix bundles core [92]. 

In 2015, a phylogenetic analysis was carried out by Leblanc et al. with the alignment of 26 sequences of biochemically or structurally characterized vHPOs and 4 sequences of bacterial non-specific acid phosphatases [90]. The phylogenetic tree revealed that vHPOs could derive from a common marine bacterial ancestor, closely related to the bacterial acid phosphatases [90]. Another clue to illustrate their phylogenetic association is the overlay of monomeric vHPOs and bacterial acid phosphatases [93]. In fact, three specific regions of vCPO from *C. inaequalis* (corresponding to structural elements and catalytic residues in the active site) were found to correspond to a conserved pattern of bacterial acid phosphatases (Figure S2). Authors indicated that many residues which matched with these conserved domains directly interact with the vanadate cofactor in the active site of vCPO [93]. The active site of these two types of enzymes seems to be very close. This observation is reinforced by the fact that vanadate is considered as an inhibitor of acid phosphatase with its ability to replace phosphate transition state. A potential PAP2-related phosphatase mechanism can be rebuilt using the active site of vCPO from *C. inaequalis* by replacing vanadate by a phosphate group [93] (Figure S2).

To determine the halide specificity of different types of vHPOs (vCPO from *C. inaequalis*, vBPO from *C. officinalis* and vIPO from *Z. galactanivorans*), their structures have been meticulously compared because no halide has been co-crystallized with these enzymes. One of the first residues highlighted in vCPO from *C. inaequalis* was the Phe397 which is replaced by a histidine in vBPO from *C. officinalis* but is present in bacterial vIPO from *Z. galactanivorans*. However, another bacterial vCPO was shown to include a histidine residue at this place [88]. Even if this residue is not involved in the catalytic mechanism, it remains unclear if it could affect the halide specificity of vHPOs [88,94]. Many studies relate directed evolution of vHPOs active site to determine the different conserved residues activity and to determine which one is responsible for halide selection. In the vCPO from *C. inaequalis*, Phe397 and Trp350 are putative halide-binding site residues by analysis of different mutants and comparison with vBPO of *C. officinalis*. More precisely, halide specificity may depend on different synergistic effects: (i) the electron density on oxygens of the vanadate cofactor, (ii) the changes of electrostatic environment near the Phe397 in mutants [95]. A more recent study also suggests that halide specificity can be explained by modification of hydrogen bond coordination and modification of the redox potential of the vanadate cofactor rather than selective halide binding. In this case, authors propose that the modification of the proximal electronic environment and of the redox potential of the vanadate cofactor may allow it to react with different halides [96].

#### 2.4.3. Enzymatic Mechanism and Specificity

**Figure 8:**
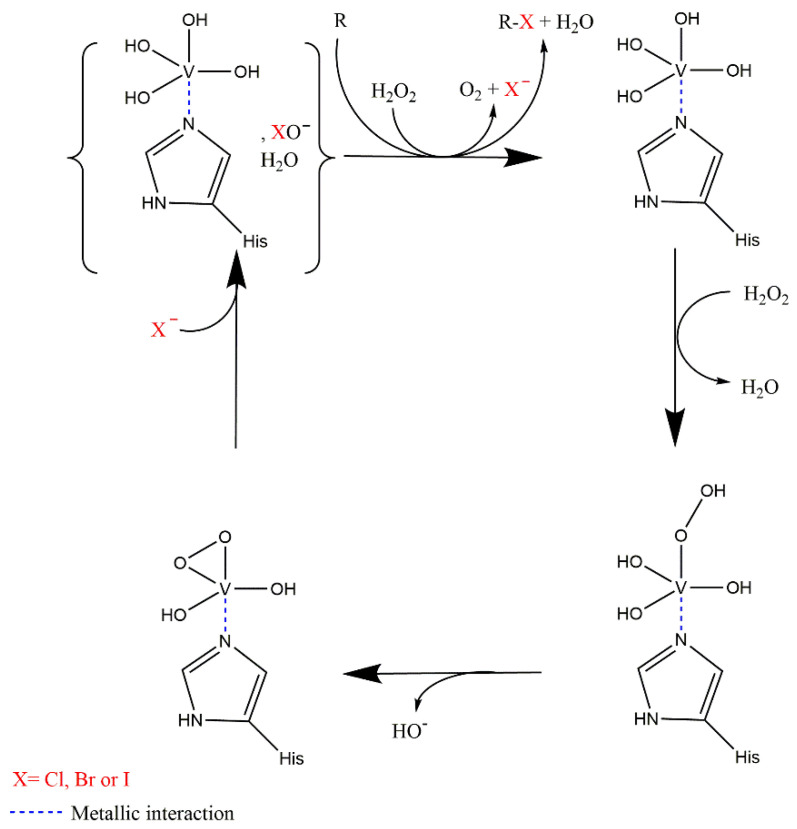
General halogenation mechanism of vanadium-dependent haloperoxidases inspired by [88].

vHPOs function is like hHPOs but with a different prosthetic group. Like hHPOs, vHPOs produce a hypohalous acid (HOX, donor of X^+^). vHPOs catalyse the two-electron oxidation of halides in presence of hydrogen peroxide. At the beginning, the vanadate cofactor at the oxidation state (V) can react with hydrogen peroxide to form a stable per-oxovanadate intermediate (Figure 8). This stable intermediate can then react with different halogens species (mainly X^−^) to form the hypohalous acid. This highly reactive hypohalous acid can then react with an acceptable substrate (R on Figure 8). Most of the studies on vHPOs suggest that this latter reaction occurs after diffusion of the hypohalous acid in a non-enzyme dependent reaction [90]. If no substrate is present, the hypohalous acid reacts with a second equivalent of hydrogen peroxide to form the corresponding halide and dioxygen.

Like hHPOs, vHPOs present a lack of selectivity during the halogenation process. Most studies did not show any control of stereo- or regioselectivity by the metalloenzymes but are in favour of a mechanism involving the diffusion of the hypohalous acid. However, this lack of selectivity is still questionable as some publications have revealed enantiospecific bromination and cyclization of sesquiterpenes using vBPO of *C. officinalis* [97]. This study of Carter-Franklin & Butler shows differences between brominated compound enzymatically obtained and brominated compound chemically obtained suggesting a certain regioselectivity of algal vBPO [98]. These differences suggest that in presence of a specific substrate, the reaction might be enzymatically controlled but if no specific substrate is present, vHPOs could unspecifically halogenate proximal molecules.

#### 2.4.4. vHPOs in Fungi

Up to now, there are 288 sequences (UniProt database) associated to fungal vHPOs but only two have been characterized, e.g., the one from *C. inaequalis* and another from *Alternaria didymospora* (previously named *Embellisia didymospora*). The first one is the only one which has been crystallized [91]. Other sequences have been only computationally detected and automatically classified as vHPOs. For computationally detected vHPOs, UniProt database is not adapted. There are a lot of duplicated and statistically unvalidated sequences and possible confusions with PAP2 enzymes can occur. To refine this result, a Blastp analysis was done on MycoCosm database using vCPO from *C. inaequalis* as query sequence [23]. For this analysis, the E-value threshold was fixed at 1.0 × 10^−50^. 105 sequences corresponded to proteins belonging to the superfamily “Acid phosphatase/Vanadium-dependent haloperoxidase” gathering approximatively 70 different fungal species with an important *Alternaria* genus cluster (including 20 different species).

The vCPO of *A. didymospora* shares 68% identity with the *Ci*vCPO [29]. This is the first characterized vHPO from a marine fungus, which has a higher affinity for bromine (Km = 5 µM) compared to chlorine (Km = 1.2 mM) at pH 5.2, this enzyme has been natively purified [29]. Another vHPO has also been detected in *Botrytis cinerea*, a fungal pathogen of plants. Authors have detected intracellular haloperoxidase activity using *o*-dianisidine assay [99]. They have shown that it was a vHPO by inactivating the protein using EDTA and reactivating it using ortho-vanadate, but the sequence of the specific protein is still not available on databases such as UniProt [99].

An interesting case is the vBPO described from the terrestrial lichen *Xanthoria parietina,* a symbiont between the green alga *Trebouxia* and an ascomycete. This study allowed the first discovery of a vBPO in a terrestrial environment. However, authors were not able to determine if the protein came from the fungal or the algal proteome, even if this enzyme shared features with algal vBPOs [100].

#### 2.4.5. Implication in Biosynthesis of Halogenated Compounds

In *C. inaequalis*, no halogenated compound has been reported to be the product of vCPO. No fungal secondary halogenated metabolites have been reported to be halogenated by a vanadium haloperoxidase so far.

#### 2.4.6. Applications and Prospect

Today, some chemistry laboratories are using the vCPO from *C. inaequalis* as part of their halogenation tools. In fact, this enzyme has been developed and marketed (heterologously or natively) to produce halogenated molecules in organic synthesis [54,57,101]. This type of enzyme has the advantage to be extremely stable in organic solvents and to produce less waste than full organic halogenation reactions. One parameter to consider in the halogenation reaction remains the pH-dependency of the biocatalyst. A recent publication reported the use of vCPO from *C. inaequalis* to form different halolactones or haloethers using a chemo-enzymatic approach. They proved that the halolactones formed using chemical reactions or chemoenzymatic reactions where comparable. However, the chemoenzymatic process was found to be more eco-friendly with less toxic solvents needed and a drastic reduction of toxic wastes as it only produced water and unreacted halide [69]. However, one of the disadvantages of this method was that the current chemoenzymatic halolactonization was not selective and produced racemic lactones. Bromocyclizations of α-and γ-allenols have been described and halofunctionalization of different alkenes by vCPO from *C. inaequalis* has also been described [102,103]. Like CPO from *C. fumago*, vCPO is able to halogenate phenols like thymol [104]. The vCPO has also been reported to be useful in the Aza-Achmatowicz reaction. This reaction allows the rearrangement of a furan ring into a dihydropyran using hypohalous acid. This reaction is frequently used in pharmaceutical biotechnologies because it provides scaffolds containing multiple functional handles for further synthetic transformations [64] (Table 2). 

### 2.5. Co-Factor-Free Haloperoxidases

#### 2.5.1. History

The third class of haloperoxidase is the cofactor-free haloperoxidase (HPOs) which mechanism does not involve any metallic cofactor. This type of haloperoxidase was discovered during the last 80s in bacteria [105]. Contrary to vHPOs and hHPOs, HPOs do not need any metallic cofactor to halogenate substrates but use a catalytic triad in the active site (Ser-His-Asp) to form the hypohalous acid [106]. 

#### 2.5.2. Structure 

So far, no fungal structure of HPOs crystallized is present in the PDB. The only structural analyses of HPOs were conducted on bacterial enzymes from *Streptomyces aurofaciens*, *S. lividians* and *Pseudomonas fluorescens*. The catalytic triad Ser-His-Asp was confirmed by site directed mutagenesis studies on the HPO from *Pseudomonas pyrrocinia* [107]. This study showed that an organic acid (acetate, benzoate or propionate coming from the reaction buffer [108]) is required to start the enzymatic reaction with the formation of a peroxoacid which allows the hydrogen peroxide activation. A tunnel inside the enzyme can bring the halide to the hydrophobic active site where it can react with a peroxoacid to form a hypohalous acid. This hydrophobic binding pocket is supposed to protect the peroxoacid against hydrolysis [106]. A structural comparison has also been carried out to determine evolutionary relationships between bacterial HPOs. This study showed structural relationships with α/β hydrolases which also harbour the catalytic triad Ser-His-Asp in their active site. Between these two enzyme classes, the central β-sheet and six helices are mostly conserved. The overall topology is similar when comparing crystal structures [109]. The HPO from *S. aureofaciens* is a monomeric protein with the central β-sheet formed by eight strands, all parallel except for the second. Strands 3 to 6 and 7 and 8 are connected by α-helices A, B, C and E. The C-terminal part is formed by helices F and G. Between strands 6 and 7, a large inclusion is formed by five helices. This general topology corresponds to the 3D structure of α/β hydrolases [110].

#### 2.5.3. Enzymatic Mechanism and Specificity

According to the mechanism described by Hofmann et al., an organic acid (coming from the reaction buffer) is required during the first step [106]. The histidine residue is hydrogen bonded to Asp and Ser at the beginning. It begins with the release of the carboxylic acid group of the organic acid bound to Ser and a water molecule. Then, hydrogen peroxide can react with this bounded acid to form a free peroxoacid which remains in the hydrophobic active site where it is attacked by halide to form hypohalous acid [106]. Then, this reactive species can freely react with an acceptable substrate to produce a halogenated metabolite. The optimal pH was described to be pH 6, as at pH 8 the organic acid can be deprotonated leading to a less effective reaction. No halide binding site was detected by analysing the structure of HPOs [106]. For halogenation, while no binding site for a substrate was described suggesting uncatalyzed reaction with the hypohalous acid species formed by the enzyme, some substrate specificity was still observed and was explained by the influence of the size and the hydrophobic environment of the active site [106].

#### 2.5.4. Detection in Fungi

No characterized or crystal structure of HPOs has been described in fungi so far. However, this class of haloperoxidases seems to be present in fungal genomes as, in UniProt database, 57 sequences related to non-heme chloroperoxidases which share features with α/β hydrolases like HPOs were computationally detected. Two sequences were found in a Mucoromycete, *Rhizophagus irregularis* and the 55 others from various Ascomycota. A Blastp conducted on MycoCosm with the HPO sequence of *Pseudomonas fluorescens* (UniProt KB-O31158 (PRXC_PSEFL)) also revealed 306 hits with an acceptable E-value (<1.0 × 10^−50^) and a percentage of identity between 66% and 49% suggesting the probable presence of HPOs in fungal genomes. 

So far, even in bacteria, where they were first described, no halogenated metabolite has been related to these enzymes.

#### 2.5.5. Prospects 

It would be interesting to isolate and characterize a fungal HPOs to study their halogenating capacity regarding the substrate nature, specificity and efficiency. We can assume that it would be probably close to hHPOs and vHPOs, which also form reactive hypohalous acid species. Such discovery would allow comparing activities for the three class of haloperoxidases from fungi. Because HPOs do not need any metallic cofactor, we could expect no oxidative destruction of the metallic cofactor causing enzymatic inactivation in comparison to hHPOs. This would be an attractive feature for biocatalysis. 

### 2.6. Flavin-Dependent Halogenases 

#### 2.6.1. History

Flavin-dependent halogenases (FDHs) are the most characterized enzymes halogenating metabolites at that time because of their presence in BGCs. Discovered during the 90s [111], FDHs are a class of enzymes which is ubiquitous across all taxa [88]. The genes encoding FDHs have been already found in fungal genomes using metagenomic analyses [112]. For example, this type of halogenating enzyme is involved in the biosynthesis of ochratoxin A in *Aspergillus carbonarius* [33]. Another example is the chlorination of radicicol, a potent heat shock protein 90 inhibitor isolated from various fungi like *Pochonia chlamydosporia* and *Chaetomium chiversii.*

#### 2.6.2. Structure

All FDHs structures described in literature have a low percentage of identity between the different kingdoms of life but 3 parts of the enzyme are necessarily present for an optimal enzymatic function. First, there is a flavin binding site where the flavin cofactor is reduced (with the help of a flavin reductase partner) which harbours the highly conserved binding motif “GxGxxG” [113]. A substrate binding pocket has also been detected in contrast to fungal haloperoxidases. This binding pocket has been described to be distant of the flavin cofactor binding pocket [114,115]. In FDHs, the hypohalite formed is not freely diffusible but it is regiospecifically delivered to the aromatic substrate via the formation of a haloamine species which is the third required part of the enzyme [88]. Here, a highly conserved lysine residue is found. It is not clear if the halide reacts with this lysine residue to form a haloamine residue or if it is just stabilized in a pocket next to this residue [116].

**Figure 9:**
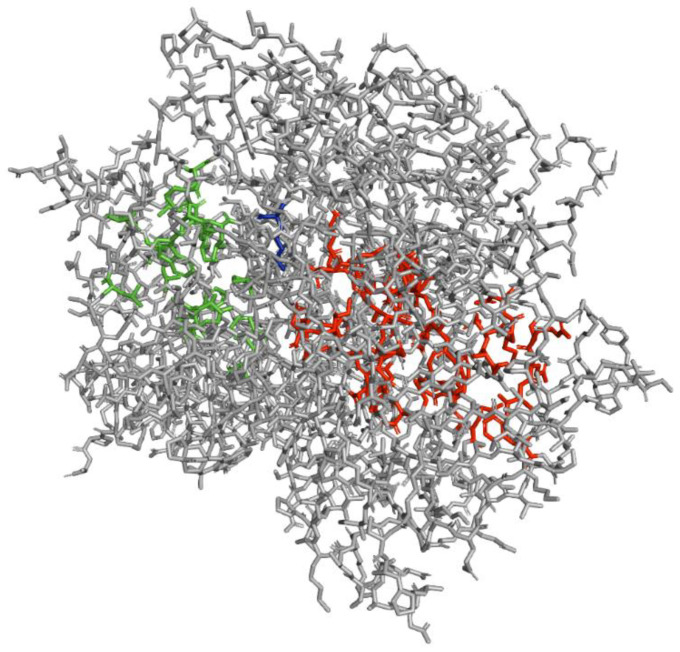
5WGR-crystal structure of wild-type MalA’, premalbrancheamide complex: separation between substrate binding pocket (green), FAD binding pocket (red), and lysine 108 (blue) [30].

In the fungal reign, only nine FDHs have been crystallized. All of them are the flavin-dependent halogenases MalA (one is the wild type, and the eight others are mutants from MalA) from *Malbranchea aurantiaca* [30]. This FDH (PDB: 5WGR) (Figure 9) allows the dichlorination of a fungal indole alkaloid, premalbrancheamide to form malbrancheamide which is a calmodulin antagonist [117].

The analysis of the protein structure shows that the active site (substrate binding pocket) and the FAD binding pocket are clearly separated [30]. The highly conserved lysine used to fix hypochlorous acid is Lys108 in MalA. As observed for the general mechanism of FDHs, premalbrancheamide undergoes electrophilic aromatic substitution with the help of Glu494 which allows an efficient binding of the substrate. The substrate is also stabilized in the binding-pocket because of the presence of a Phe496 which facilitates a favourable hydrophobic interaction with the aromatic ring of premalbrancheamide [30] (Figure 9).

#### 2.6.3. Enzymatic Mechanism and Specificity

**Figure 10:**
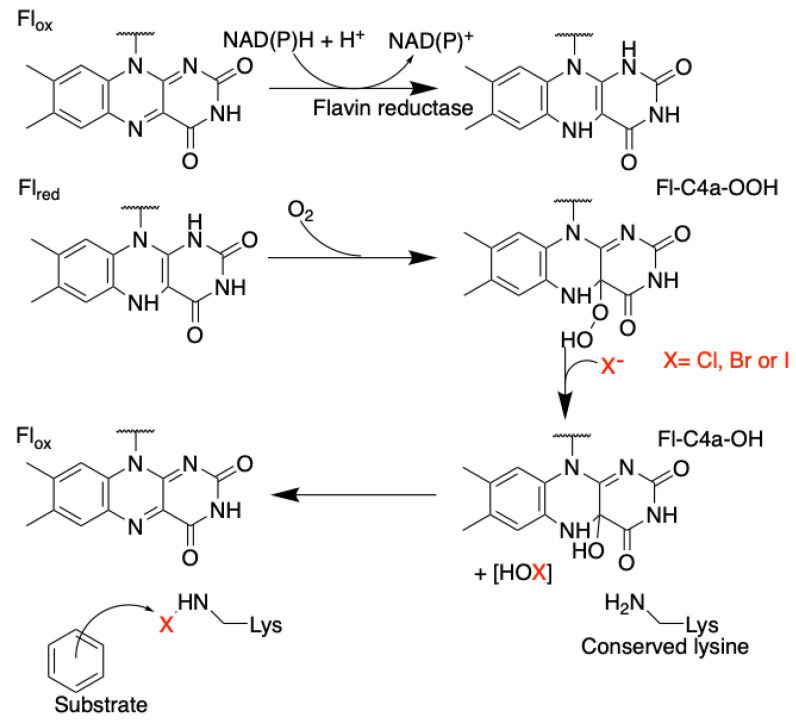
FDH schematic reaction mechanism inspired by [88].

Flavin-dependent halogenases catalyse the 2e^−^ oxidation of the halide (X^−^) to the hypohalite (OX^−^) [88] (Figure 10). A consensus mechanistic scheme based on the biochemical characterization of L-tryptophan FDH (FDHs using L-tryptophan as substrate: PrnA from *Pseudomonas fluorescens*, RebH from *Lechevalieria aerocolonigenes* and PyrH from *Streptomyces rugosporus*) describes today the general mechanism of FDHs in fungi [88,118].

The mechanism closely resembles the one described for the flavin-dependent oxygenases. First, there is a reduction of the flavin cofactor by a 2e^−^ transfer from NAD(P)H (+H^+^) catalyzed by a flavin reductase. The flavin-oxidized (Fl_ox_) cofactor is reduced in the flavin cofactor (Fl_red_) in the FAD binding pocket. It is possible that the flavin reductase is encoded in the BGC with the other genes required for the synthesis of the metabolite, this reaction partner does not seem to be specific to FDHs [119]. Then, the reduced cofactor (Fl_red_) can react with molecular oxygen (O_2_). A transient species is formed: Fl-C(4a)-OOH [88]. This transient species is considered to be a “OH^+^” donor which can hydroxylate the proximally bound aromatic substrate via electrophilic aromatic substitution or it can react with halide to form hypohalous acid [118]. This hypohalous acid species (X^+^ donor) travels through a tunnel to another conserved pocket close to the bonded substrate where it can react with the highly conserved active lysine residue, or it can be stabilized through halogen bonds with the amine [115,116]. The bound substrate can then undergo electrophilic aromatic substitution with the bound HOX species to form the halogenated molecule. This step is possible thanks to the presence of a closer glutamine which stabilizes the substrate.

The formation of the three flavin-cofactor states has been spectroscopically (UVs) detected in FDHs. It is noteworthy that those two independent reactions take place in FDHs. First, the formation of the highly reactive hypohalous acid occurs with the reduction of the flavin cofactor and its attack by molecular oxygen (O_2_). The halogenation step follows. As the two actives sites are spatially separated in the enzyme, the hypohalous acid must be translocated from one to the other. The main hypothesis for this migration is that there is a tunnel between the two active sites inside the enzyme. Then, the enzyme controls the fixation of the substrate to catalyse a regiospecific halogenation [120].

#### 2.6.4. FDHs in Fungi and Their Implication in the Biosynthesis of Metabolites

In fungi like in other organisms such as bacteria, the biosynthesis of many secondary metabolites is encoded by BGCs which contain all the genes required for the effective enzyme production involved in their biosynthesis. Contrary to haloperoxidases, all fungal flavin-dependent halogenases (FDHs) described have been found as part of BGCs, facilitating their association to halogenated metabolites [121]. In terms of substrates, FDHs have been shown to halogenate many different structures with reactive carbons such as indole, phenol [38] or pyrrole [122]. While some are able to directly halogenate free substrates [123], most FDHs strictly prefer substrates bound to an acyl carrier protein (ACP).

##### Fungal FDHs Halogenating Indole Structures

**Figure 11:**
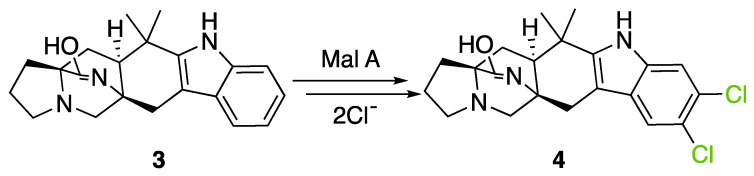
Premalbrancheamide (**3**) chlorination by MalA to obtain malbrancheamide (**4**).

A basic search of fungal flavin-dependent halogenases in the UniProt first highlighted 696 sequences. 15 of those detected proteins have already been characterized in the literature (Table 1). The remaining proteins have only been computationally detected but not characterized. Among the 15 described proteins, we find the previously mentioned FDHs MalA from *Malbranchea aurantiaca,* which is responsible for the biosynthesis of the indolic alkaloid malbrancheamide (**4**) (Figure 11) [117]. A mechanism has been recently proposed by Fraley et al. to describe the formation of malbrancheamide (**4**) by MalA where the chlorine is stabilized by the formation of a chloramine intermediate bounded with Lys108 as previously described for the general mechanism [30]. The main difference here is the late-stage halogenation of premalbrancheamide (**3**) compared to L-tryptophan FDHs where halogenation usually takes place at the beginning of the biosynthesis. However, the general mechanism remains the same as described before. Further investigation with a Blastp analysis on MycoCosm using MalA sequence revealed the presence of 277 analogue proteins in fungal genomes, with an E-value lower than 1.0 × 10^−50^ and a percentage of identity ranging from 38% to 77%. This shows that many more remain to be discovered with potentially new related halogenated compounds.

##### Fungal FDHs Halogenating Phenol Structures

**Figure 12:**
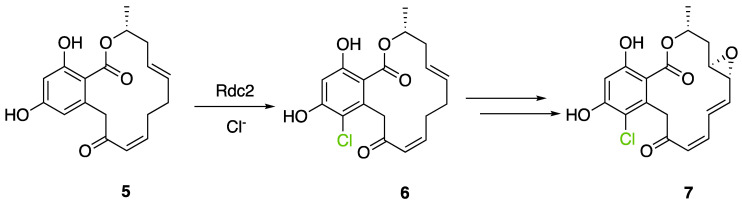
(*R*)-Monocillin II (**5**) chlorination by Rdc2 to form pochonin D (**6**), precursor of radicicol (**7**).

The first flavin-dependent halogenase (Rdc2) identified in fungi was discovered in 2008 by Reeves et al. in *Pochonia chlamydosporia.* No crystalized structure for this enzyme was obtained but a homology model was built to analyse its enzymatic mechanism. Interestingly, the binding site of this enzyme allows the fixation of the natural substrate (*R*)-monocillin II (**5**) to form pochonin D (**6**) and to obtain the final oxidised product radicicol (**7**) (Figure 12) [31].

**Figure 13:**
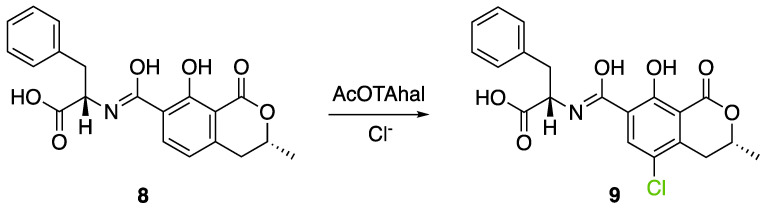
The FDH AcOTAhal catalyzes the chlorination of ochratoxin B (**8**) to form ochratoxin A (**9**) in *Aspergillus carbonarius* [33].

It also allows the loading of a wide range of other macrolactones such as isoquinolines (4-hydroxyisoquinoline and 6-hydroxyisoquinoline) which were reported to be halogenated by Rdc2 [71]. This enzyme contrasts with the more specific FDHs previously described using L-tryptophan as physiological substrate because of their lower substrate selectivity. Nonetheless, like other FDHs described, Lys74 is strictly conserved and essential for the enzyme catalysis and the stabilization of the highly reactive halogen species by the formation of a chloramine. Lys74 is present in the N-terminus hydrophobic region where the substrate binds. The chlorination occurs in *ortho* of the hydroxyl group in the three cases ((*R*)-monocillin II and 4- or 6-hydroxyisoquinoline) [71]. A homologous enzyme was found in the radicicol-producing fungus *Chaetomium cheversii* and named radH (Table 1). [32,70]. A Blastp analysis performed on MycoCosm using radH sequence and an E-value threshold of 1.0 × 10^−50^ revealed 2300 hits with a percentage of identity from 37% to 79%, suggesting a frequent presence of radH homologues in fungal genomes. Because we found more hits than the number of genomes available (2008), it can be supposed that some species may possess multiple copies of radH-like proteins in their genome. According to databases (UniProt or MycoCosm), the most detected FDHs in fungi are the radH-like flavin-dependent halogenases. It is present in a lot of *Aspergillus* species (Figure S3), which can be explained by the fact that this genus has been extensively studied in relation with the production of the chlorinated mycotoxin ochratoxin A (**9**) (OTA), leading to its important presence in fungal sequence databases such as MycoCosm. OTA is a food mycotoxin which is highly controlled because of its important toxicity for humankind [33]. For this reason, many studies have targeted the ochratoxin A BGC. A radH-like flavin-dependent halogenase [32,70] named AcOTAhal has been described in 2016, which catalyses the chlorination of ochratoxin B (**8**) (OTB) (the non-halogenated form of OTA) to form OTA (Figure 13) [33]. The main producers of OTA are *Aspergillus ochraceus* and *Penicillium verrucosum* but more largely the *Aspergillus* and *Penicillium* species [124].

**Figure 14:**
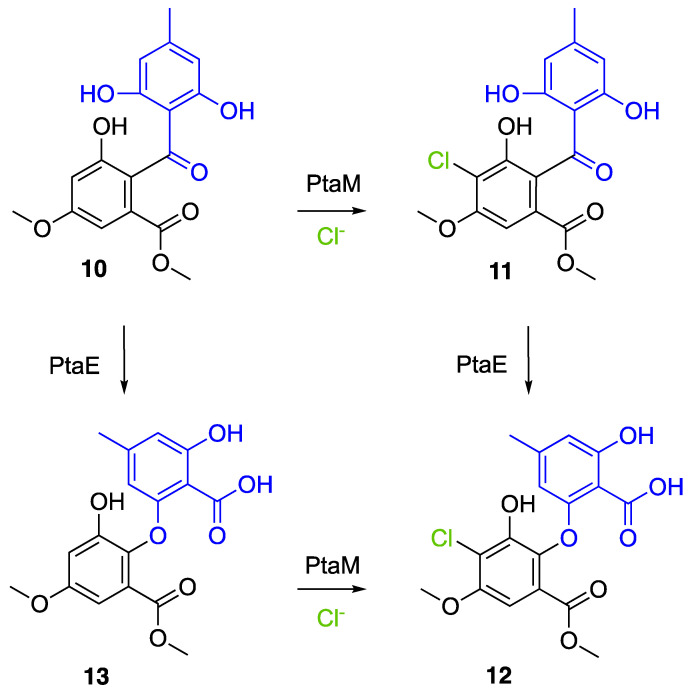
Chlorination of isosulochrin (**10**) by PtaM in *P. fici* to obtain chloroisosulochrin (**11**), a precursor of pestheic acid (**12**). Isosulochrin (**10**) can be oxidized by PtaE to form RES-1214-1 (**13**), which is chlorinated by PtaM to form pestheic acid (**12**) in *Pestalotiopsis* sp.

Another FDH-containing BGC has been described in the plant endophyte *Pestalotiopsis fici*, which is responsible for the biosynthesis of pestheic acid (**12**), one of the precursors of chloropupukeananes (compounds showing antimicrobial, antitumor and anti-HIV activities). In the BGC, *ptaM* encodes a flavin-dependent halogenase which is a homologous to the radH protein with a conserved FAD binding domain. The FDH is essential for the production chloroisosulochrin (**11**) (Figure 14). A knock-out mutant was created on *ptaM* revealing that the production of chloroisosulochrin (**11**) was abolished whereas the nonchlorinated precursor isosulochrin (**10**) was accumulated indicating that the *ptaM* gene is essential for the chlorination step (Figure 14) [35]. In an alternative way, isosulochrin (**10**) can be oxidized by PtaE to obtain RES-1214-1 (**13**) before being halogenated by PtaM to obtain pestheic acid (**12**) in *Pestalotiopsis* sp. [35].

**Figure 15:**
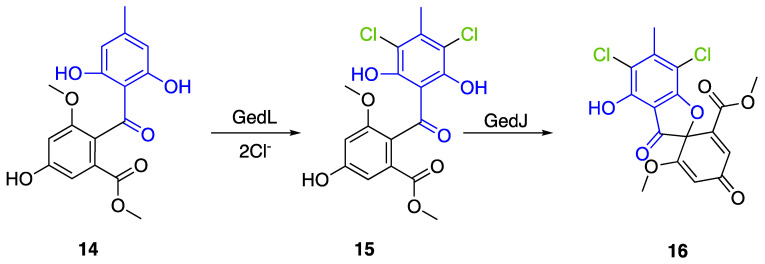
Dichlorination of sulochrin (**14**) by GedL in *A. terreus* to form dihydrogeodin (**15**) in the biosynthesis of geodin (**16**).

Geodin (**17**) is a chlorinated metabolite produced by diverse fungal species and in particular *Aspergillus terreus* where the BGC has been described. The BGC involves a FDH, GedL, for the dihalogenation step of sulochrin (**14**) in dihydrogeodin (**15**) (Figure 15). Contrary to PtaM on isosulochrin, the halogenation catalyzed by GedL on sulochrin occurs on the other phenolic moiety, showing the interesting regioselectivity of this type of enzyme. The BCG description has been realized after heterologous reconstitution of the geodin gene cluster from *A. terreus* in the expression platform *Aspergillus nidulans* [36]. Geodin has been described to enhance fibrinolytic activity of vascular endothelial bovine cells [125].

**Figure 16:**
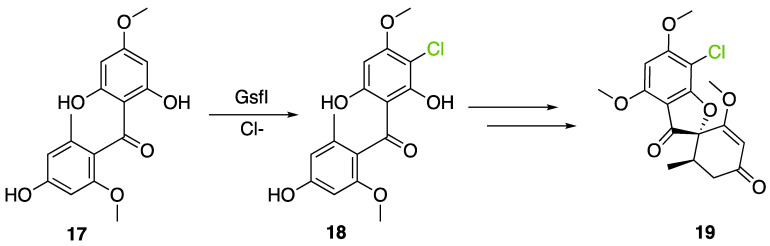
Chlorination of griseophenone C (**17**) by GsfI to form griseophenone B (**18**), precursor of griseofulvin (**19**) in *P. aethiopicum*.

The well-known antifungal polyketide, griseofulvin (**19**), also involves a flavin-dependent halogenase during its biosynthesis by *Penicillium* species (*P*. *griseofulvum*, *P. chrysogenum* or *P. aethiopicum*). In *P. aethiopicum*, the griseolfulvin gene cluster was investigated and a gene, GsfI, was characterized to produce a halogenase that shares high similarity (60%) with the flavin-dependent chlorinase radH (Figure 16). To confirm its implication in the griseofulvin biosynthesis, the gene was disrupted. LC-MS analysis showed abolition of the griseofulvin production together with an accumulation of the non-chlorinated precursor, griseophenone C (**17**) proving the implication of GsfI in the griseofulvin biosynthesis [37,126].

**Figure 17:**
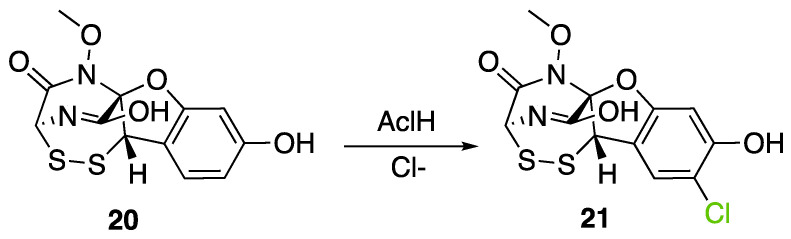
Chlorination of dechloroaspirochlorine (**20**) by AclH in *A. oryzae* to form aspirochlorine (**21**).

Another FDH has been described in *Aspergillus oryzae* by analysing the biosynthesis of the mycotoxin aspirochlorine (Figure 17). The *acl* BGC contains one halogenase AclH which catalyses the chlorination of the aspirochlorine precursor, dechloroaspirochlorine (**20**). Aspirochlorine (**21**) production was completely abolished by deleting the AclH gene. Complementation was done in the mutant *A. oryzae* ΔaclH with the *aclH* gene, leading to the restoration of aspirochlorine production.

**Figure 18:**
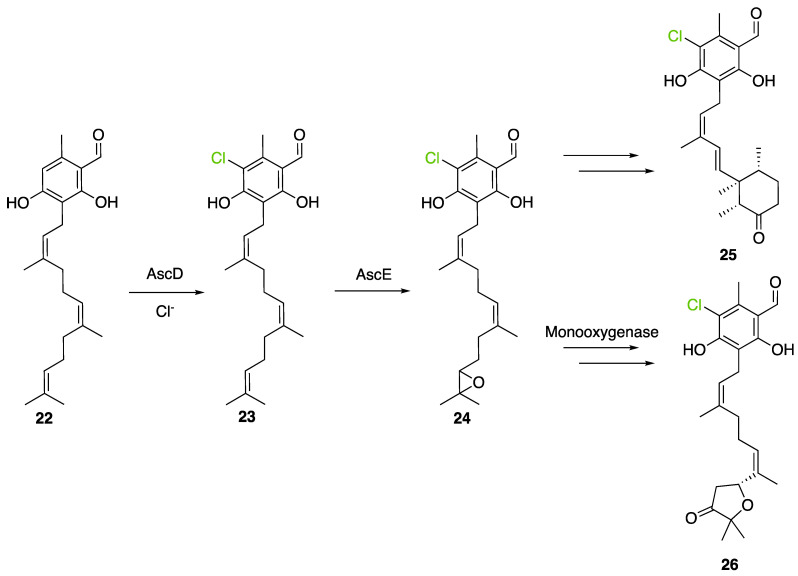
Chlorination of ilicicolin B (**22**) by AscD in *A. egyptiacum* to form ilicicolin A (**23**) and then the epoxide form (**24**) in the biosynthesis of ascochlorin (**25**) and ascofuranone (**26**).

Another FDH, AscD, has been described in the BGC of ascofuranone (**26**) and ascochlorin (**25**) in the fungus *Acremonium egyptiacum* (Figure 18). These two molecules are meroterpenoids possessing diverse bioactivities including anticancer anti-trypanosomiasis. Halogenation process takes place at the beginning of the biosynthesis, with ilicicolin B (**22**) being chlorinated to obtain ilicicolin A (**23**) which, after several steps, leads to ascofuranone (**26**) or ascochlorin (**25**). Ilicicolin A is then oxidised by AscE, an epoxidase, to obtain ilicicolin A epoxide (**24**). From this precursor two routes have been described: the molecule is hydroxylated by the monooxygenase AscH leading to ascofuranone, if not, it leading to the cyclohexanone ascochlorin [39].

**Figure 19:**
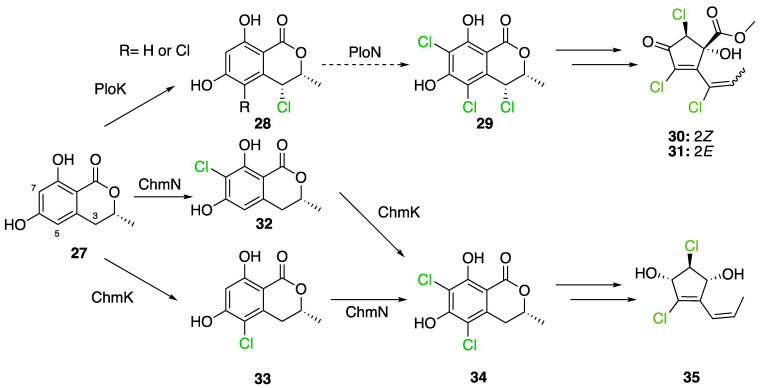
Halogenation of (*R*)-6-hydroxymellein (**27**) by PloK and PloN in *L. palmae,* and in *H. velutinum* by ChmK and ChmN. If R = H, (2*R*,3*R*)-3-Chloro-6-hydroxymellein, if R = Cl, (2*R*,3*R*)-3,5-Dichloro-6-hydroxymellein (**28**). (2R,3R)-3,5,7-Trichloro-6-hydroxymellein (**29**), palmaenone A (**30**), palmaenone B (**31**). (*R*)-7-Chloro-6-hydroxymellein (**32**), (*R*)-5-chloro-6-hydroxymellein (**33**), (*R*)-5,7-dichloro-6-hydroxymellein (**34**), and cyclohelminthol IV (**35**).

Cyclohelminthols and palmaenones are specialized metabolites respectively produced by *Helminthosporium velutinum* and *Lachnum palmae* which carry chlorine atoms on an oxygenated cyclopentene system. These two types of metabolites, even if they are produced by different organisms, share the same biosynthetic intermediate, 6-hydroxymellein (**27**). The two BGCs have been described for cyclohelminthol (*chm*) and palmaenone (*plo*) [41]. Genes encoding tryptophan halogenases homolog proteins have been detected in each BGC: *chmK* and *chmN* in *H. velutinum* and *ploK* and *ploN* for *L. palmae.* But if *chmK* and *chmN* are found in the same BGC, *ploN* is found outside the cluster in *L. palmae*. Halogenases from each BGC have been cloned into *A. oryzae* to analyze their activities. In the two cases, halogenation of 6-hydroxymellein (**27**) is the first step followed by undescribed modifications to obtain cyclohelminthols IV (**35**) and palmaenones A (**30**) and B (**31**) (Figure 19) [41].

For palmaenone biosynthesis, PloK first catalyzes mono or dichlorination on C3 and C5 and then PloN (66% homolog to ChmN) catalyzes the third chlorination on C7. For cyclohelminthol biosynthesis, ChmK catalyzes chlorination on C5 and ChmN on C7 but without particular order [41]. These observations again highlight the high regioselectivity of FDHs enzymes.

**Figure 20:**
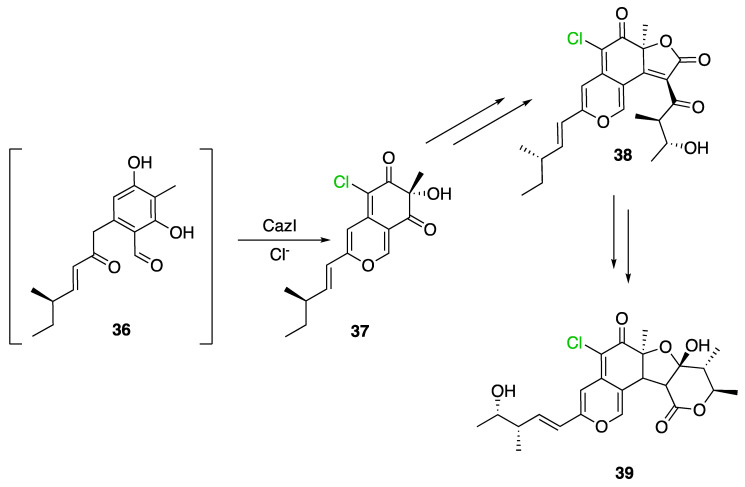
Chlorination of the biosynthetic intermediate (**36**) to obtain cazisochromen (**37**) in the biosynthetic pathway of chaetoviridin A (**38**) and chaetomugilin A (**39**).

Identification and description in *Chaetomium globosum* of chaetoviridin and chaetomugilin BGC led to the discovery of the FDH CazI. Genetic inactivation studies were performed to describe the involvement of each *caz* genes in the biosynthesis of these metabolites (Figure 20). Chlorination takes place on an unstable biosynthetic intermediate (**36**) to form cazisochromen (**37**). Then, the biosynthetic route continues to finally obtain chaetoviridin A (**38**) and later chaetomugilin A (**39**) [42].

##### Other Non-Classical Fungal FDHs

FDHs were also detected in the honey fungus *Armillaria mellea* [40]. This basidiomycetous tree pathogen produces a large variety of structurally related toxins known as melleolides. While it was obvious that some of these secondary metabolites undergo a chlorination step during their biosynthesis, the characterization of the melleolides gene cluster in 2016 did not show any FDH encoding gene [40]. A flavin-dependent oxidoreductase was present, but it lacked the strongly conserved motif GWxWxxPL of FDHs. Authors have then identified 5 genes encoding putative FDHs in the *A. mellea* genome but outside the melleolides BGC (armH1 to armH5). To evaluate their implication in the chlorination of melleolides, these five genes were cloned, and proteins were expressed heterologously in *E. coli*. The unchlorinated melleolide F was given as free substrate to test their halogenation specificity and the final product was analysed by HPLC-HR-ESIMS. The authors showed that all the five enzymes were able to chlorinate melleolide F to form 6′-chloro-melleolide F, but based on the peak areas, armH4 showed the best results. Bromination was then also successively conducted with armH4, but iodination assays failed to produce iodinated metabolite. So far, these are the only studies performed to understand the chlorination of melleolides. Further studies involving disruption of the FDHs genes, would probably give more confidence in the identification of the halogenating enzyme of melleolide F. However, this study shows that two different groups in the fungal flavin-dependent halogenases can probably be distinguished: those which accept free substrates and those which are more specific and need substrates bounded to a carrier-protein. We do not have enough data yet to assume that this is a general trait of fungal halogenases [40].

**Figure 21:**
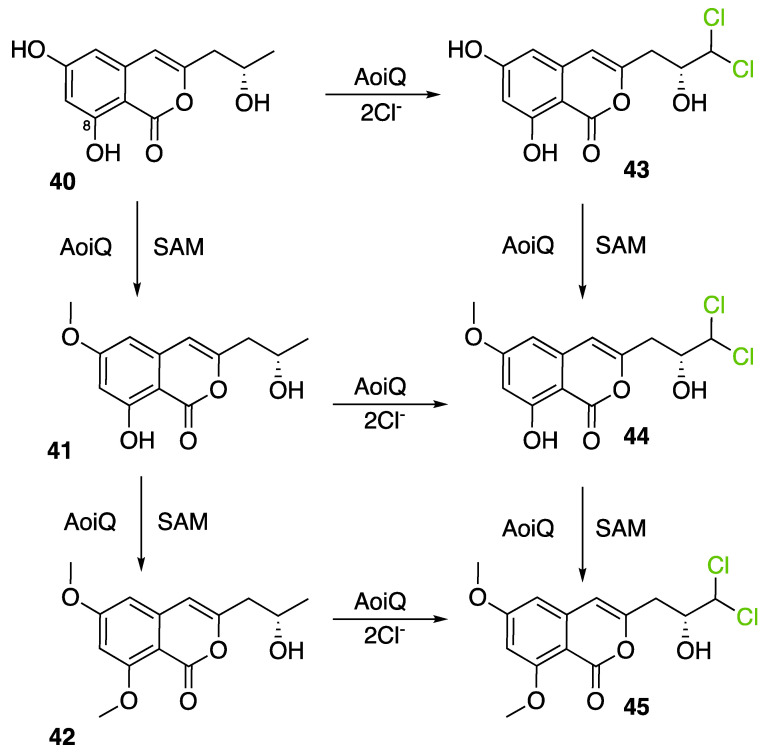
Biosynthetic route examples of the hybrid enzyme AoiQ described in *Aspergillus oryzae* on diaporthin derivatives de-*O*-methyldiaporthin (**40**), diaporthin (**41**), 8-methoxyldiaporthin (**42**), de-*O*-methyl-dichlorodiaporthin (**43**), dichlorodiaporthin (**44**), and 8-methoxyl-dichlorodiaporthin (**45**).

More recently, a multitask FDH from *Aspergillus oryzae* (AoiQ) was shown to be able to mono or dihalogenate a non-activated carbon atom on an alkyl chain or perform several methylations [127]. This particular enzyme has been detected and characterized in the genome of *Aspergillus oryzae* RIB40 encoded by the AoiQ gene. It corresponds to a 1014 amino acids protein (2 times bigger than standard FDHs) with two different parts. At the N-terminus part, flavin-dependent halogenase conserved motifs are present (flavin cofactor binding site and tryptophan binding site) while at the C-terminus part of the enzyme, a conserved S-adenosyl methionine (SAM) binding domain is found. This feature is typically present in methyltransferases (detailed hereafter 2.7). Blast homology research made by Chankhamjon et al. highlighted that homolog enzymes can be found in at least a dozen of other fungal genomes [127]. AoiQ protein was shown to be responsible for the introduction of a geminal dichloro moiety on diaporthin derivatives De-*O*-methyldiaporthin (**40**), diaporthin (**41**) and 8-methoxyldiaporthin (**42**) in *Aspergillus oryzae* (Figure 21). This enzyme is the only fungal aliphatic halogenase described so far. An example of the biosynthetic routes involving AoiQ can be observed in Figure 21. Halogenation can occur once or twice leading to de-*O*-methyl-dichlorodiaporthin (**43**), dichlorodiaporthin (major product) (**44**) and 8-methoxyl-dichlorodiaporthin (**45**). Monochlorinated compounds can also be dimethylated by the AioQ SAM part of the enzyme [127].

#### 2.6.5. Application and Prospect

Today, the use of flavin-dependent halogenases in biocatalysis is still somewhat limited because of their lack of stability, the low diversity of substrates they can handle and the high difficulty to produce them in large scale [128]. To improve the flavin-dependent halogenase efficacy, different engineering steps have been proposed. For example, the use of cross-linked enzyme aggregates (CLEAs, glutaraldehyde) has been successfully reported in literature to stabilize the halogenase RebH from *Lechevalieria aerocolonigenes* [129] which is today the most used flavin-dependent halogenase in biocatalytic halogenation [113,130]. Other studies also reported the directed evolution of FDHs to improve their thermostability [131] or extend their substrates specificity [132]. The modified enzymes were then successfully used in chemical synthesis to generate small halogenated molecules such as chlorinated alkaloids or antibiotics derivatives (ex: chloropacidamycin) [133,134]. So far, the main applications of FDHs in biocatalysis were reported for recombinant Rdc2/RadH which allowed successful reactions on similar structures as its natural substrates but also on other skeletons such as curvularin, curcumin or isoquinoline (Table 3) [70,71]. The MalA enzyme has also been studied for its potential in biocatalysis on fungal indole alkaloids, which display a large range of biological activities. The chemoenzymatic way to produce these molecules allowed better selectivity and yield [135]. As for the AoiQ enzyme, which discovery is still somehow quite recent, no further applications have been described so far, but it looks promising. Indeed, we can imagine that regioselective halogenation on non-activated aliphatic carbons would be of interest for biocatalysis.

### 2.7. Non-Heme Iron-Dependent Halogenases

#### 2.7.1. Mechanism

Another specific class of halogenases exists in nature, namely the non-heme iron-dependent halogenases (NHFe halogenases) such as WelO5 discovered in 2014 in the cyanobacteria *Hapalosiphon welwitschii* [136]. Up to now, no three-dimensional nor characterized structure of a NHFe fungal halogenase has been published in protein databanks. However, some putative sequences in fungal genomes could correspond to this class of halogenating enzymes, which mechanism of action and reactivity could be of interest to chemists.

**Figure 22:**
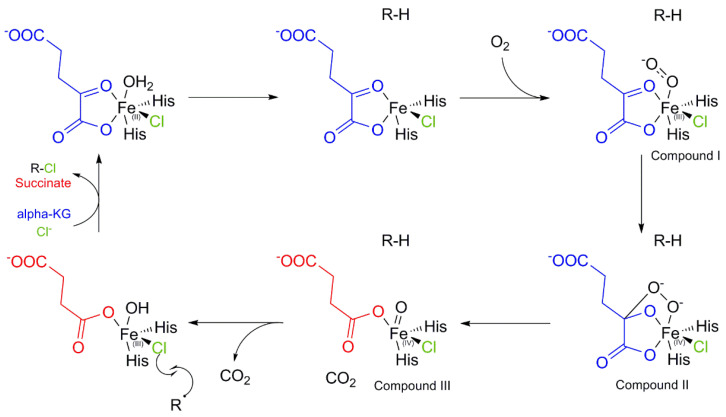
Catalytic cycle of NHFe halogenase inspired by Butler et al. [75].

Indeed, NHFe halogenases allow halogen (mainly chlorine) incorporation by using radical chemistry on unactivated aliphatic chains [88] and closely mirrors that of NHFe hydroxylases. First, the organic substrate is fixed in the enzyme active site which triggers a modification in the resting state and allows the coordination of dioxygen by Fe_(II)_. Then a “Hanauske-Abel and Günzler mechanism” of the Fe_NH_-αKG oxygenases occurs leading to hydrogen-atom abstraction by the haloferryl species (X-Fe_(IV)_ = O) [137]. The halogenase mechanism proceeds by a rebound attack by the halogen atom at the iron centre (Fe_(III)_-X) on the organic radical substrate to form the halogenated organic compound and the release of Fe_(II)_ [75] (Figure 22).

#### 2.7.2. Detection in Fungi

In UniProt database, 24 putative sequences corresponding to NHFe halogenases were found in fungi, but none has been analysed and characterized in the literature. All these sequences have been computationally detected. A Blastp analysis on MycoCosm using the WelO5 protein from *Hapalosiphon welwitschii* [138], with an E-value threshold of 1.0 × 10^−50^ revealed no hit in fungal genomes. In the same way, no more result was obtained when using the syrB2 NHFe halogenase from *Pseudomonas syringae* [139].

#### 2.7.3. Prospect

This review of the literature and the databases shows that this enzyme class needs to be more studied in fungi to better assess their occurrence and functions. In other kingdoms like bacteria or algae, NHFe halogenases are able to halogenate substrates bound to a carrier protein. They can also halogenate free small molecules [136]. The presence of this enzyme class in fungal genomes is questionable because no hit was obtained using bacterial NHFe halogenases as query sequences in Blastp analysis. Of course, not all fungal genomes were investigated and there can still exist such enzymes in fungi, especially regarding the 24 putative sequences found on UniProt database, which is more complete compared to the recent database MycoCosm. However, compared to other classes of halogenating enzymes reviewed so far, NHFe halogenases seem to be—if really present in fungi—very rare or very different from bacterial NHFe halogenases. Moreover, first attempts of application of NHFe enzymes in biocatalysis have raised many issues as the activity of heterologous NHFe halogenase remained low and the substrate needed to be bound to a specific carrier protein to be usable [140].

### 2.8. S-Adenosylmethionine-Dependent Halide Methyltransferase

#### 2.8.1. Mechanism

**Figure 23:**
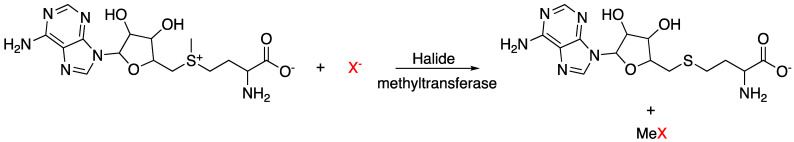
Example of a halide methyltransferase catalyzed reaction.

Another class of halogenating enzyme, the SAM-dependent halide methyltransferase has also been described in nature, such as the HOL protein produced by *Arabidopsis thaliana* [141]. However, they are not well documented in the literature, in particular in fungi. This enzyme class allows the biosynthesis of halomethanes “CH_3_-X” which can potentially act a donor of halide for the biosynthesis of halogenated secondary metabolites [88,142]. SAM-dependent halide methyltransferases allow the formation of MeCl, MeBr and MeI (F^−^ is not accepted). These enzymes catalyse nucleophilic substitution reactions between SAM and halide ions to form methyl-halides (Figure 23).

#### 2.8.2. Detection in Fungi

No crystal structure of SAM-dependent halide methyltransferase is available in the fungal reign, but some have been detected for example in the *Hymenochaetaceae* family [142]. However, it could be proposed that the previously mentioned multitask FDH enzyme named AoiQ (Section 2.5.4), identified from *A. oryzae* could also be placed into this class as conserved SAM binding domain was observed in this enzyme, and the type of halogenation observed on non-activated aliphatic carbon was very different from classical FDHs.

#### 2.8.3. Prospects

Much remains to be understood concerning this enzyme class regarding their mechanism, metabolic role and their implication in the biosynthesis of halogenated secondary metabolites. Further studies on the enzyme AoiQ may also reveal more insights into this category of halogenation enzymes.

### 2.9. Summary of Fungal Halogenation Enzymes

Enzymatic halogenation in fungi has been widely studied (Figure S3). Different halogenating enzyme classes exist but lots of grey areas remain in the different protein families and their enzymatic mechanism. One example is given by the few numbers of three-dimensional structures available for each enzyme class. Most of the time, only one 3D structure is available for one enzymatic class. It is then impossible to compare fungal enzyme structures. However, other studies on enzymatic halogenation in other kingdoms can be useful to formulate hypotheses.

Another aspect is the association between halogenated metabolites and their halogenation enzyme. Lots of enzymatic classes responsible for halogenation are unspecific. For example, haloperoxidases seem to produce halogenated reactive species but do not control the regioselectivity or the stereoselectivity of the halogenation. Moreover, their occurrence in genomes have not been found to be related to BGCs, precluding the possibility to make correlations between these enzymes and specific metabolites. However, in bacteria some vHPOs have been recently found to be specifically related to BGCs and to be involved in the production of napyradiomycin and merochlorins A-D [143,144]. We do not know yet if this type of halogenating enzyme is mostly implicated in the secondary metabolism or in other functions. This association is more easily performed for halogenated secondary metabolites involving flavin-dependent halogenases which are more specific and mainly present in BGCs, except for melleolides.

So far, no enzyme able to fluorinate specific or unspecific substrates has been described in fungi. The occurrence of fluorinated natural products is very rare in nature even if some fluorinating enzymes have been described in bacteria like 5’-fluoro-5’-deoxyadenosine synthase in *Streptomyces cattleya* [145]. Even if fluorine has a great abundance in nature, its incorporation scarcity in secondary metabolites is probably due to its extreme electronegativity. On the other hand, the number of iodine containing compounds is also very low in number because of the low abundance of iodide in nature [5].

The *Natural Products Atlas* referenced 865 halogenated molecules isolated from fungi in early 2021 [7]. These molecules either possess a single halogen in their structure or several. Some of the molecules described even possess the three types of halogens studied here, namely bromine, chlorine and iodine. Among all these metabolites, only those previously cited in this review have had their biosynthesis associated to a halogenating enzyme. For some of them, they were even completely described. In accordance with what has been said previously, we mainly find metabolites arising from an FDH, with the exception of only one metabolite, caldariomycin, for which halogenation is claimed to be catalyzed by a hHPO. In comparison with the total number of halogenated metabolites described in fungi (Figure 1), it means that knowledge on relationships between halogenated metabolites and their halogenating enzyme occurring in these organisms are still poorly resolved.

## 3. Halogenated Metabolites

### 3.1. Methodology

**Figure 24:**
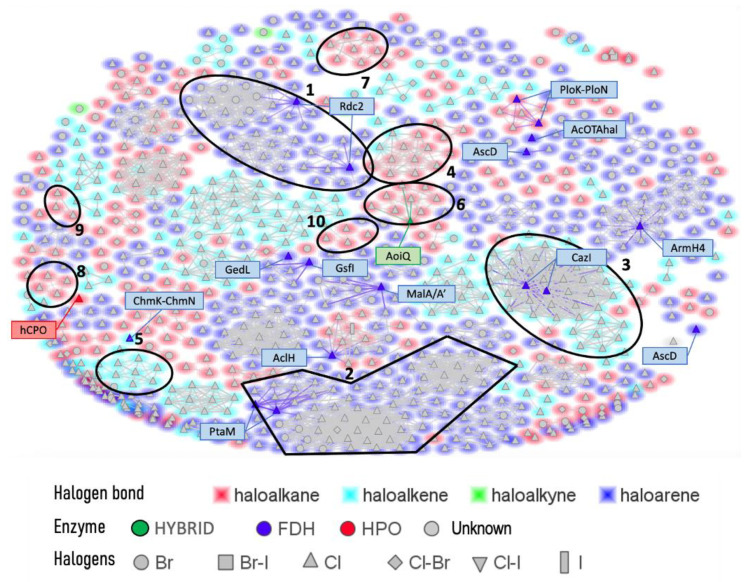
Similarity molecular network of the 865 halogenated fungal metabolites described in the *Natural Product Atlas* in 2021. This plot was created with DataWarrior software, where structures were clustered based on their similarity using the “FragFp” descriptor (set to 80% for similarity analysis). Each structure is represented by a marker whose shape is defined by the halogens present: chlorine (△), bromine (○), iodine (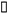), chlorine and bromine (◇), chlorine and iodine (▽), and bromine and iodine (□). Structures for which a halogenating enzyme was described as being involved in their biosynthesis appeared as colored: in green for hybrid enzyme, in blue for flavin-dependent halogenases (FDH), and in red for haloperoxidases (HPO). Finally, the type of halogen bond for each molecule is represented by a halo surrounding the marker, which is blue for haloarenes, turquoise for haloalkenes, red for haloalkanes, and green for haloalkynes. Classification of the structures according to their halogen bonds was performed using the classyfireR package on R Cran [147] and the Chebi descriptors. Black and numerated circles (1 to 10) correspond to studied and detailed clusters in the present review.

Further investigation of these 865 halogenated fungal metabolites was performed using the open source DataWarrior software to investigate their chemical similarities and differences [146]. A molecular network was constructed using the default “FragFp” descriptor, allowing to filter the molecular structures and link them together according to their similarities (Figure 24). The DataWarrior’s default descriptor FragFp is a substructure fragment-based binary fingerprint. It relies on a dictionary of 512 predefined structure fragments. This descriptor can be used to calculate similarities between molecules. The FragFp similarity between two molecules is the number of fragments that both molecules have in common divided by the number of fragments being found in any of the two molecules. Typically, chemists will consider molecules to be structurally close if their similarity value is about 0.90 or above. The constructed network which included all fungal halogenated metabolites available in the NP Atlas database allowed us to explore the chemical diversity found in fungal halogenated metabolites. Moreover, for each compound, information about 1) the type of halogens present (Cl, Br, I, F), 2) the halogenating enzyme described and 3) the type of halogen bond was added to the list (using the automated chemical classification package “ClassyFire” on R Cran [147]). These three filters allowed to highlight meaningful information on the network and different hypotheses could be raised about halogenation enzyme classes being involved for the biosynthesis of many compounds.

Some clusters group together structures, including metabolites for which the halogenation enzyme is described (Figure 24). In many cases, they correspond to biosynthetic intermediates or molecules of the same class, which only differ by the position of chemical groups (regioisomers). However, in some cases, molecules that were linked to a molecule for which the halogenating enzyme is known, arose from different biosynthetic pathways or fungal organisms. From this point, it was possible to put forward the hypothesis that, given the strong similarity of the structures, these metabolites might be halogenated by the same type of enzyme. As most halogenating enzymes known to be involved in the biosynthesis of secondary metabolites correspond to FDHs, mainly reacting on indole or phenol systems, this approach was mostly applied to compounds in the haloarene class. We can see that most clusters of haloarene-type compounds are related to known halogenation enzymes belonging to FDHs, which is consistent with the reactivity of this type of enzyme. On the other hand, for haloperoxidase enzymes, the use of the network occurred to be very limited, especially as the only metabolite, which is halogenated by a heme haloperoxidase, caldariomycin (**2**) (classified as a haloalkane) was isolated in the network and not linked to any other metabolite. It was therefore difficult here to make any hypotheses for this type of enzyme. Moreover, it is not clear yet if and how these enzymes are involved in the biosynthesis of secondary metabolites. Given to their usual non-inclusion into BGC, it can be anticipated that they can halogenate many different compounds. However, no evidence of this type has been reported so far, except for the bacterial compounds napyradiomycin and merochlorins A-D [143,144], which could be classified as haloalkenes and haloalkanes. Further studies on fungal HPOs are then needed to answer these questions.

### 3.2. Compounds in the Haloarene Class

**Figure 25:**
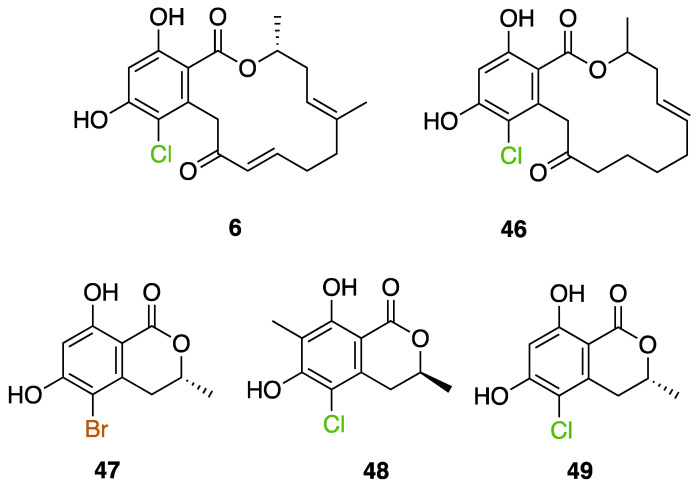
Structures of the metabolites found in the pochonin D cluster 1: pochonin D (**6**), monorden E (**46**), palmerin D (**47**), chaetosemin G (**48**), and 5-chloro-6-hydroxymellein (**49**).

In cluster 1, the pochonin D (**6**) produced by *Pochonia chlamydosporia*, has been described to be halogenated by the flavin-dependent halogenase Rdc2 [56]. Using the descriptor FragFp, a high number of halogenated fungal metabolites showed a similarity score greater than 0.85 (on a scale of 0 to 1). This means that the fragments present in these molecules are very similar to those found in molecule **6.** In addition, the halogen atom is systematically found in the *ortho* position of a hydroxyl group on the aromatic ring (both chlorine and bromine atoms can be found). Among these molecules with a high similarity index, we found molecules belonging to the pochonin family which are produced by the same fungus, but also halogenated molecules produced by other fungal species such as monorden E (**46**) produced by *Humicola* sp. [148], palmerin D (**47**) produced by *Lachnum palmae* [149], chaetosemin G (**48**) produced by *Chaetonium* sp. [150] and 5-chloro-6-hydroxymellein (**49**) produced by an unknown strain [151] (Figure 25).

Thus, if we rely on the strong structural similarity between these halogenated molecules, particularly in terms of the position of the halogen on the molecule, we can assume that the halogenating enzymes involved in their biosynthesis may be related or very close to Rdc2, the previously reported FDH halogenating pochonin D (**6**). 

If we analyse more precisely the structure of the molecule in correlation with the described biosynthetic pathway of pochonin D (**6**), the precursor of radicicol, we can observe that the cyclisation of the polyketide chain occurs before the halogenation by the enzyme (here Rdc2) [56]. On the non-halogenated precursor (R)-monocillin II (**5**), two positions are then available for halogenation. Interestingly, it is the less reactive position on C6 that undergoes halogenation. Indeed, position C4 is located between two hydroxyl groups on the aromatic ring which makes it more reactive (by intramolecular electrostatic effects) than position C6 (Figure 12). It also seems easier to access for possible spontaneous reaction in view of the steric hindrance. Therefore, by extension and in view of the similar structures of the four molecules mentioned above, involvement of a flavin-dependent halogenase of the Rdc2 family in their biosynthesis seems to be a consistent hypothesis.

This hypothesis is reinforced by the genomic analysis previously carried out on MycoCosm [23] in the search for fungal strains that possess genes in their genome that are homologous to the RadH protein (like Rdc2). It has been shown that there is a strong presence of “RadH like proteins” in the fungal genomes available in the database. More specifically, if we look at the four molecules mentioned above for which the halogenation enzyme is not known, for one of their producers, *Humicola* sp., one related genome could be found in MycoCosm (*Thermomyces lanuginosus* SSBP, previously *Humicola lanuginosa*). In this available genome, we could find a putative protein that matched with a flavin-dependent halogenase of the “radH like protein” type with a homology percentage of 60%. This protein could hypothetically be involved in the biosynthetic pathway of monorden E (**46**).

**Figure 26:**
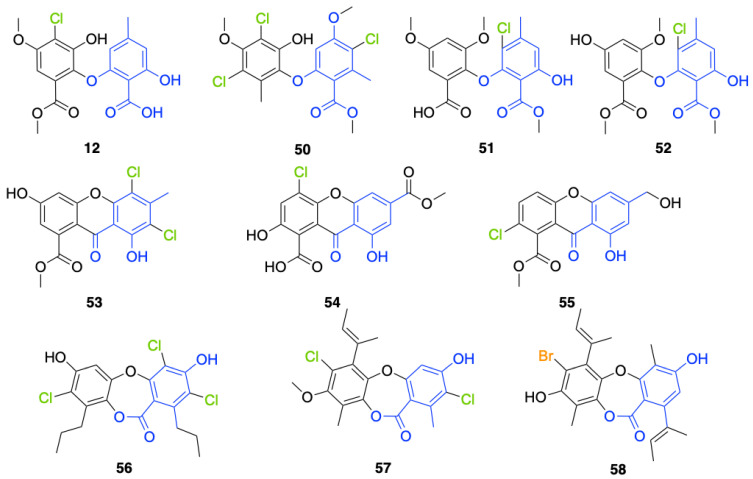
Pestheic acid (**12**) is included in cluster 2 containing buellin (**50**) from *Diploicia* sp.; chrysine D (**51**), penicillither (**52**), and penicillixanthone (**53**) from *Penicillium* sp.; 4-chlorocurvularinic acid (**54**) from *Curvularia* sp.; engyodontiumone B (**55**) from *Engyodontium* sp.; spiromastixone N (**56**) from *Spiromastix* sp.; aspersidone (**57**) from *Aspergillus* sp.; and 7-bromofolipastatin (**58**) from *Aspergillus* sp.

The construction of the network proved useful for hypothesising potential metabolites that could be halogenated by FDHs in view of the similarities in structure and specificity of these enzymes. Another cluster can be shown to illustrate this overall idea, the cluster 2 which contains the diphenyl ether compound pestheic acid (**12**) known to be halogenated by a the FDH PtaM and produced by *Pestalotiopsis* sp. (Figure 14) [152]. This structure closely resembles to buellin (**50**) isolated from the lichen *Diploicia canescens* [153], chrysine D (**51**) and penicillither (**52**) both isolated from *Penicillium* species [154,155]. Again, similar structures produced by different organisms may probably be halogenated by similar enzymes. At least 67 molecules are grouped together in cluster 2 and they share the same skeleton with two aromatic rings linked by an ether bond. Some compounds present a cyclised pyran-4-one moiety between these two aromatic rings, forming xanthone-type compounds such as penicillixanthone (**53**) [155], 4-chlorocurvularinic acid (**54**) [156] and engyodontiumone B (**55**) [157]. In the centre of this cluster, many metabolites possess an extra ester bond forming typical depsidones, like spiromastixone N (**56**) [158], aspersidone (**57**) [155] and 7-bromofolipastatin (**58**) [159] (Figure 26). Research of PtaM homologous proteins showed no result for *Diploicia*, *Curvularia* and *Spiromastix* genera in the available 2 genomes on MycoCosm (*Helicocarpus griseus* UAMH 5409 and *Cochliobolus lunatus* m118 v2.0). On the contrary, *Penicillium* sp. and *Aspergillus* sp. returned 26 and 352 hits respectively, with protein similarities from 49% to 70% of identity for *Penicillium* sp. and from 45% to 69% for *Aspergillus* sp. These latter genera could then possess homologous proteins to PatM with similar functions. 

### 3.3. Compounds in the Haloalkene Class

**Figure 27:**
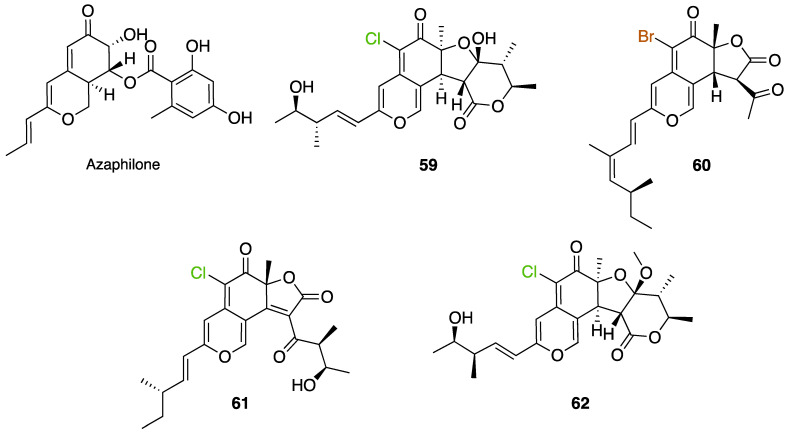
Representative azaphilone-type metabolites from cluster 3: 11-epichaetomugilin A (**59**) from *Chaetomium* sp.; 5-bromoochrephilone (**60**) from *Penicillium* sp.; chaetoviridin E (**61**) and chaetomugilin B (**62**) from *Chaetomium* sp.

In the molecular network some other clusters of metabolites appeared, but, this time, corresponding to compounds in the haloakene class. For example, a fairly large cluster (cluster 3, Figure 27) of about thirty molecules linked halogenated metabolites derived from azaphilones (pigments in *Monascus* sp.) [160]. BGCs described for this type of compounds have revealed the presence of typical FDH sequences such as the CazI previously described and hypothesized to chlorinate the bicyclic core of chaetomugilin A (**39**) and chaetoviridin A (**38**), metabolites belonging to the family of chaetoviridin E (**61**) and chaetomugilin B (**62**) [42,161].

These metabolites, if we refer to the biosynthesis of azaphilones, are derived from the polyketide pathway coupled with a fatty acid pathway [162]. An esterification between the polyketide chromophore and the β-keto acid forms the backbone of the molecule. Here, the clustering was consistent as almost all molecules in this cluster were similar with their halogen atom (chlorine/bromine) positioned on the ⍺-carbon of the ketone (α,β-unsaturated ketones).

It should be noted that this position is highly reactive because it is vicinal of a carbonyl group. Many ketones undergo electrophilic ⍺-substitution reactions since the hydrogens in this position are more labile. Thus, halogenation reactions on this reactive ⍺-carbon are either acid or base catalyzed and can even be autocatalytic when releasing acid as a product. The mechanism of this reaction involves the keto-enolic equilibrium with the formation of enol or enolate species. However, the presence of an α-β double bond can disturb the formation of enolates [163]. In fact, halogenation of a α,β-unsaturated ketone is usually performed in organic chemistry by using strong oxidants such as OXONE^®^ and hydrohalic acid [164] or metachloroperbenzoic acid (pKa 7.57, a weak acid) in DMF in the presence of HCl at 25 °C. As most structures in this cluster correspond to α,β-unsaturated ketones, with the halogen in ⍺-position, we can again assume it is catalyzed by specific FDH enzymes, closely related to the CazI enzyme.

**Figure 28:**
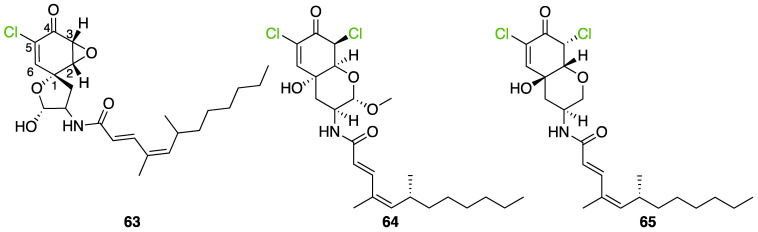
Metabolites present in cluster 4, aranochlor A (**63**) from *Pseudoarachniotus* sp., dankastatin A (**64**) and B (**65**) from *Gymnascella* sp.

Another cluster (cluster 4) can be associated to the previous one as it also highlighted azaphilone-type molecules. These latter showed chlorine on both sides of the ketone leading one of the halogen bonds being on a *sp*^3^ carbon (and considered as a haloalkane on Figure 28). These molecules such as aranochlor A (**63**) [165], dankastatin A (**64**) and B (**65**) present a much longer carbon chain with an amide bond linking the heterocycles to the carbon chain [166]. Considering the similar bicyclic core, with halogenation taking place in ⍺ of a ketone, we can hypothesize that the enzymes implied to halogenate those compounds might be similar to cluster 3, at least for position C-5. For the second chlorine on position C-3, another mechanism could be involved, especially as we can see that derivatives of this family of compounds correspond to C-2-C-3 epoxy derivatives as for aranochlor A (**64**). Indeed, epoxides are very sensitive to nucleophilic additions, leading halogenation of these species to be quite easy [167]. Thus, spontaneous formation of this halogen bond in C-3 from an epoxide intermediate could also be an option for these compounds. 

**Figure 29:**
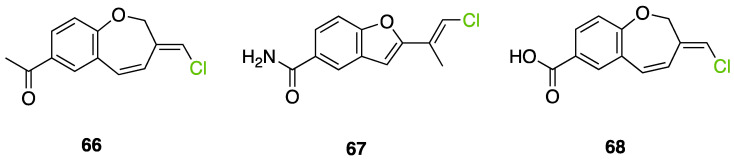
Some metabolites from cluster 5: pterulone (**66**), calocerin (**67**), and 2,3-dihydro-1-benzoxepin derivative 4a (**68**).

Interesting metabolites from basidiomycetes can be found in cluster 5 like pterulone (**66**) produced by *Pterula* sp. (Figure 29) [168]. Theses metabolites carry a chlorine atom on a terminal double bond which is unusual. This cluster gathers 9 metabolites like calocerin B (**67**) or 2,3-dihydro-1-benzoxepin derivative 4a (**68**) both produced by *Favolaschia* sp. [169]. The halogenation takes place on a *sp*^2^ carbon, but no enzyme described before seems to be able to perform this type of reaction. Indeed, FDHs and haloperoxidases catalyze electrophilic halogenation. Here, the total synthesis of pterulone (**66**) has been described using the way of nucleophilic addition. A Wittig reaction was used to introduce chlorine on a ketone using (chloromethyl)triphenylphosphonium chloride with n-butyllithium in THF at 0 °C, which corresponds to harsh chemical conditions [170]. So far, no enzyme performing this kind of reaction in fungi has been described to selectively halogenate ketones by displacing an oxygen atom. Further studies on the biosynthetic pathways of these compounds would be needed to further understand the way they are naturally halogenated.

### 3.4. Compounds in the Haloalkane Class

With the exception of caldariomycin (**2**) and the very particular diaporthin derivatives (**43**, **44** and **45**) mentioned above, all clusters for which a halogenating enzyme was described corresponded to haloarenes or haloalkene molecules with a FDH involved. From this observation, molecules classified as haloalkanes (or alkyl halides) occurred to be of interest considering their halogenation mechanism. In fact, preparation of alkyl halides in organic chemistry usually requires the use of radical reactions on alkanes or alkenes, with reagents such as *N*-bromosuccinimide (NBS), *N*-chlorosuccinimide (NCS), Br_2_ or Cl_2_ and energy such as light or heat. The formation of the carbon-halogen bonds on *sp*^3^ carbons can be difficult and require harsh chemistry. If these types of bonds are present in nature, they might be catalyzed by enzymes. In relation with what was previously presented, non-heme iron-dependent halogenases (NHFe) and S-adenosylmethionine-dependent halogenases (SAM-H) are the two types of halogenation enzymes that are capable of carrying out halogenations on non-activated aliphatic chains, using radical or nucleophilic mechanisms. However, these enzymes are poorly known and described in fungi. This is why a closer look into the structures in the haloalkane class was carried out. 

**Figure 30:**
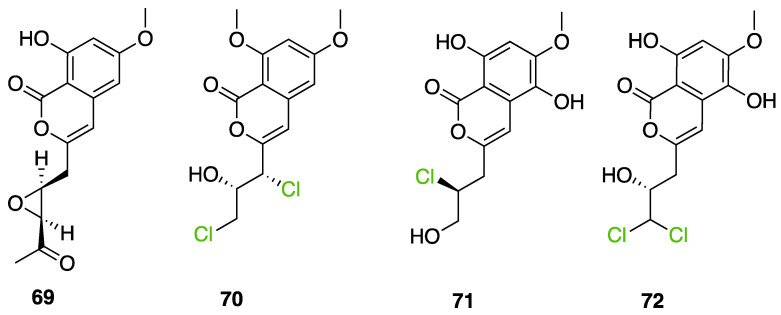
Metabolites displaying chlorination on aliphatic chains in the studied cluster 6, peniisocoumarin C (**69**) from *Penicillium commune*, (9R)-8-methyl-9,11-dichlorodiaportin (**70**) from *Hamigera fusca,* and peniisocoumarin F (**71**) and J (**72**) from *Penicillium commune*.

Among molecules classified as haloalkanes, the previously mentioned diaporthin derivatives could be retrieved in cluster 6, which gathered 9 molecules produced by different species of fungi such as *Hamigera* sp., *Ampelomyces* sp., *Penicillium* sp. and another undescribed species (Figure 30). They correspond to different isocoumarins classified as haloalkanes. The halogen atoms are found on the aliphatic chains carried by the molecules. AoiQ, which is a particular multitask enzyme with both FDH and SAM motifs, is involved in the biosynthesis of (**43**), (**44**) and (**45**) as explained above. Dichlorodiaportin (**44**) is dichlorinated by this enzyme but also methylated to obtain the methoxy groups. Regarding the similarity of structure here, it can be assumed that a similar protein is involved in the biosynthesis of (9*R*)-8-methyl-9,11-dichlorodiaportin (**70**) [171], peniisocoumarin F and J (**71**, **72**) [172] and all the metabolites present in this cluster with halogenated unactived aliphatic chains. 

If no enzyme could be clearly proposed in this kind of structure, halogenation on aliphatic chains seem to be complicated with weak reactivity. However, all molecules present a hydroxyl group on the carbon in α position. This suggests the possible spontaneous formation of halogen bonds here from an epoxide intermediate again. In fact, peniisocoumarin C (**69**), which was isolated together with peniisocoumarins A-B and D-J from *Penicillium commune* actually corresponds to the proposed intermediate allowing good reactivity for spontaneous halogenation on that position [173,174].

**Figure 31:**
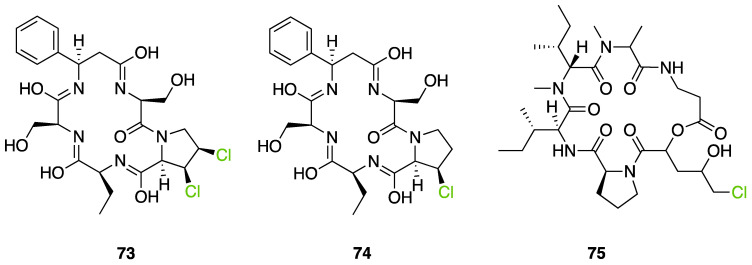
Example of halogenated non ribosomal peptides found in this specific cluster 7, cyclochlorotine (**73**) and cyclochlorotine B (**74**) from *Penicillium islandicum*, destruxin-A4-cyclohydrin (**75**) from unknown fungi.

Further considering haloalkane-type molecules, cluster 7 attracted our attention as it included halogenated secondary metabolites belonging to the class of non-ribosomal peptides, described from strains of the genera *Penicillium* sp., *Talaromyces* sp., *Beauveria* sp. and an unknown strain (Figure 31). Once again, halogens are placed on saturated carbon chains and therefore on unreactive positions. In this case, we can refer to the cyclochlorotine (**73**) biosynthesis reported in 2016 [175]. Considering the chlorine atoms on the molecules, the authors suggested the incorporation of a 3,4-dichloroproline during the formation of the peptide. However, they could not find any sequence coding for a halogenase in the BGC of cyclochlorotine (**73**). Despite their attempts to identify elsewhere in the genome the halogenase responsible for the formation of the unusual Pro(Cl_2_), by searching for conserved signatures of NHFe halogenases and α-ketoglutarate dependent halogenases, they could not detect any. They also hypothesized the combined action of a dehydrogenase and a FDH, but overexpression experiments of candidate genes were not conclusive. Finally, one recent study on cyclochlorotine (**73**) BGC in *P. islandicum* found that **73** is dichlorinated by a tailoring process involving an enzyme named CctP2, which can halogenate unactivated carbons. This enzyme was described to share no homology with any known halogenases, leading it to be a new type of enzyme that activates inert C-H bonds like NHFe halogenases [176].

For monochlorinated compounds in this cluster, while spontaneous hydrohalogenation involving hydrohalic acid (HCl, HBr) [177] on a double bond can still be considered as an explanation for their formation, we can assume similar halogenation mechanisms inside a family of compounds.

To finish, as already shown, halogenation of alkanes is well represented in fungi. NHFe halogenases and SAM-dependant halide methyltransferases (like AoiQ) are the only known enzymes that can halogenate unactivated aliphatic carbons. However, they are poorly described in fungi. And yet, many fungal compounds carry halogen on aliphatic chains as evidenced in clusters 8, 9 and 10.

**Figure 32:**
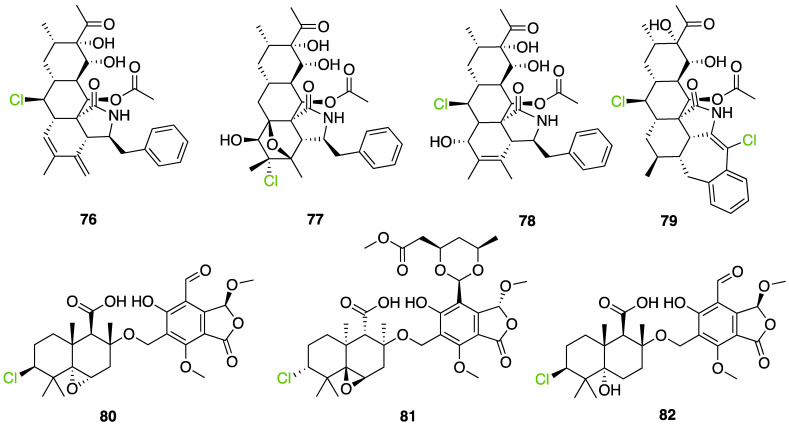
Compounds belonging to cluster 8: curtachalasin derivatives L (**76**), I (**77**), and K (**78**) and xylarichalasin A (**79**); and cluster 9: pestalotiopens A (**80**), B (**81**), and C (**82**).

Cluster 8 is composed of curtachalasin L (**76**), I (**77**) and K (**78**) together with xylarichalasin A (**79**) produced by *Xylaria* sp. [178,179] (Figure 32). All these metabolites carry halogenations on cyclohexane rings. Interestingly, during 12-epi-fischerindole G formation catalyzed by the bacterial NHFe halogenases WelO5 in *Hapalosiphon welwischii*, halogenation of a cyclohexane has been described [138]. We can then hypothesise that a similar protein is produced by this fungus and involved in the biosynthesis of these compounds. This hypothesis can be applied for metabolites in cluster 9 where halogenation again occurs on the cyclohexane ring of the three metabolites pestalotiopens A (**80**), B (**81**) and C (**82**) produced by *Pestalotiopsis* sp. [180] (Figure 32). Our search for WelO5 homologous proteins in Xylariales genomes available on MycoCosm gave no result. However, it is possible that even if a fungal NHFe halogenase was present, its sequence might be too different from bacterial NHFe halogenase WelO5.

**Figure 33:**
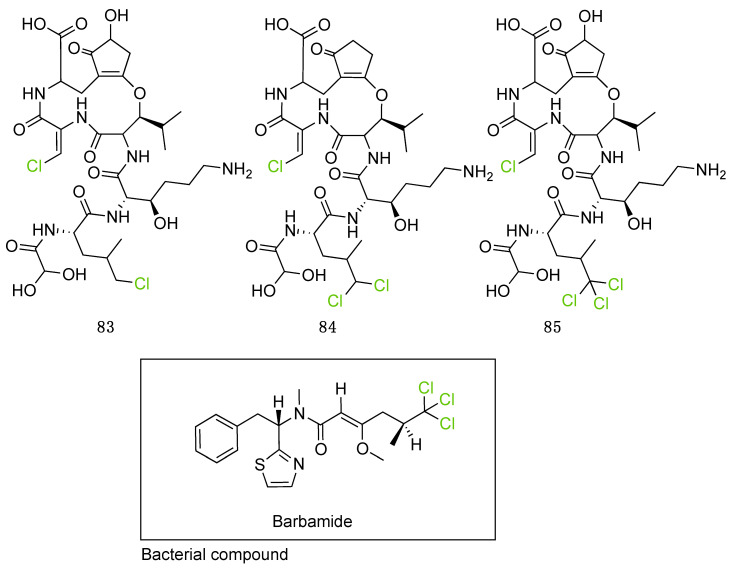
Cluster 10: victorins B (**83**), D (**84**), and E (**85**) from *Cochliobolus victoriae* and bacterial compound barbamide.

Cluster 10 is composed of the ribosomally synthesized and post-translationally modified peptides (RiPPs) victorins B (**83**), D (**84**) and E (**85**) from *Cochliobolus victoriae* [181,182,183] (Figure 33). All these molecules present terminal chlorines on aliphatic chains. The chlorinated lipopeptide barbamide described in the literature from the marine cyanobacterium *Lyngbya majuscula* present the same trichlorinated carbon sp^3^ as **84**. Biosynthesis of barbamide has been investigated and was shown to be performed by a hybrid NRPS/PKS enzyme. In the corresponding biosynthetic cluster, barB1 and barB2 encode two proteins that share homology with NHFe halogenases SyrB1-SyrB2 and CmaB from *Pseudomonas syringae*. These enzymes have been described to halogenate the amino-acid L-threonine for SyrB2 and L-alloisoleucine for CmaB before their insertion into non ribosomal peptides (NRP) [139,184]. For barbamide, trichlorination of leucine was then shown to be catalyzed by BarB2, when leucine is bound to BarB1 following the NHFe halogenase radical mechanism [184]. In the case of **83**, **84** and **85**, halogenation could be due to the presence of a protein belonging to this same enzymatic family. Indeed, halogenation also occurs on a leucine residue, and we can hypothesize a similar reaction on the amino acid before its insertion in the different peptides. However, ribosomes are described to insert only canonical amino acids in peptides making this hypothesis questionable. Kessler et al. who have worked on victorin biosynthesis hypothesized that NHFe halogenases could be involved but no homolog enzyme is found in the genome of the fungi *C. victoriae*. In fact, difference between fungal and bacterial NHFe halogenases could be important [183].

## 4. Conclusions

Fungi are great factories of specialised metabolites which can be promoted in many fields such as therapeutics, food industry, agronomy and cosmetics. Fungal secondary metabolism has been widely studied over the last decades and has led to the characterization of numerous enzymatic families, and in the case of this review, of different families of halogenating enzymes. Some are easier to characterize than others because of their specific position in the fungal genome in BGCs such as most of the FDHs. However, for others such as haloperoxidases for example, their characterization remains poor. All classes of halogenating enzymes discovered so far allow to explain much of the wide diversity of halogenated metabolites found in fungal metabolomes. Nevertheless, as reviewed, not many halogenated compounds reported from fungi have had their biosynthesis and more particularly their halogenation mechanism solved. Much remains to be discovered in this field. Regarding the high diversity of halogenated metabolites found in fungi, the diversity of skeletons on which halogen atoms are incorporated also revealed to be beyond the knowledge we have on fungal halogenating enzymes so far.

## Figures and Tables

**Table 1:** Halogenation enzymes characterized in fungi in 2021.

Enzyme Class	Entry *	Protein Names	Organism	Strain	Ref.
Heme-dependent haloperoxidase (EC 1.11.1.10)	P04963	Chloroperoxidase **CPO**	*Leptoxyphium fumago* *(Caldariomyces fumago)*		[27]
Vanadium-dependent haloperoxidases (EC 1.11.1.10)	P49053	Vanadium chloroperoxidase **vCPO**	*Curvularia inaequalis*		[28]
	P79087 (unreviewed)	Vanadium chloroperoxidase **EdCPO**	*Alternaria didymospora* *(Embellisia didymospora)*		[29]
Flavin-dependent halogenases (EC 1.14.14.-)	L0E155	Flavin-dependent halogenase **malA**	*Malbranchea aurantiaca*		[30]
	B3FWT7	Non-heme halogenase **rdc2**	*Metacordyceps chlamydosporia* *(Pochonia chlamydosporia)*		[31]
	C5H881	Non-heme halogenase **radH**	*Floropilus chiversii* *(Chaetomium chiversii)*		[32]
	A0A1R3RGJ2	Ochratoxin halogenase **OTAhal**	*Aspergillus carbonarius*	ITEM 5010	[33]
	A2R6G7	Ochratoxin halogenase **ota5**	*Aspergillus niger*	CBS 513.88/FGSC A1513	[34]
	A0A067XMV4	Flavin-dependent halogenase **ptaM**	*Pestalotiopsis fici*	W106-1/CGMCC3.15140	[35]
	Q0CCX4	Sulochrin halogenase **GedL**	*Aspergillus terreus*	NIH 2624/FGSC A1156	[36]
	D7PI14	Halogenase **gsfI**	*Penicillium aethiopicum*		[37]
	Q2UPC7	Flavine halogenase **aclH**	*Aspergillus oryzae*	ATCC 42,149/RIB 40	[38]
	A0A455R7M0	Flavine halogenase **ascD**	*Acremonium egyptiacum* *(Oospora egyptiaca)*		[39]
	G3FLZ7	Flavin-dependent halogenase **armH1**	*Armillaria mellea*		[40]
	G3FLZ8	Flavin-dependent halogenase **armH2**	*Armillaria mellea*		[40]
	A0A0U2JT80	Flavin-dependent halogenase **armH3**	*Armillaria mellea*		[40]
	A0A0U3AL34	Flavin-dependent halogenase **armH4**	*Armillaria mellea*		[40]
	A0A0U3C228	Flavin-dependent halogenase **armH5**	*Armillaria mellea*		[40]
	N/A **	Flavin-dependent halogenase **chmK**	*Helminthosporium velutinum*	yone96	[41]
	N/A **	Flavin-dependent halogenase **chmN**	*Helminthosporium velutinum*	yone96	[41]
	N/A **	Flavin-dependent halogenase **ploK**	*Lachnum palmae*	NBRC 106495	[41]
	N/A **	Flavin-dependent halogenase **ploN**	*Lachnum palmae*	NBRC 106495	[41]
	N/A **	Flavin-dependent halogenase **CazI**	*Chaetomium globosum*		[42]

* In UniProt database. ** N/A: Not applicable. Corresponds to FDHs not found in databases, but described in the literature.

**Table 2:** Examples of biocatalytic reactions reported in the literature using haloperoxidases (*Cfu*CPO: *C. fumago* heme-dependent chloroperoxidase, *Ci*vCPO: *C. inaequalis* vanadium-dependent chloroperoxidase, r: recombinant protein, n: native protein).

Reaction	Substrate	Enzyme and Reagents	Product	Refs.
Sulfoxidation	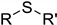	r*Cfu*CPO + H_2_O_2_	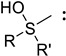	[59]
Epoxidation	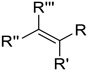	r*Cfu*CPO + H_2_O_2_	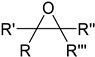	[59]
Halohydroxylation of alkene	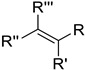	n*Cfu*CPO or r*Ci*vCPO + H_2_O_2_ + X^−^	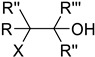	[60]
Halogenation of phenol	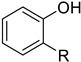	nvHPO or rhHPO + H_2_O_2_ + X^−^	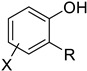	[61]
Oxidation of indole	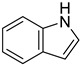	n*Cfu*CPO + H_2_O_2_	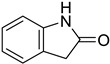	[62]
Oxidation of pyrrole	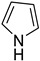	n*Cfu*CPO + H_2_O_2_	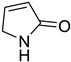	[62]
Achmatowicz reaction	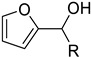	rvHPO or rhHPO + H_2_O_2_ + X^−^	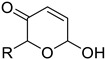	[63,64]
Flavone and flavanone halogenation	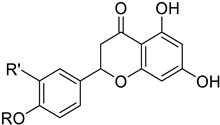	n*Cfu*CPO + H_2_O_2_ + X^−^	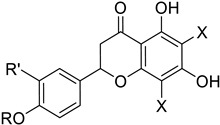	[65]
Halogenation of barbituric acid and derivatives	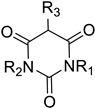	n*Cfu*CPO + H_2_O_2_ + X^−^	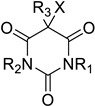	[66]
Halohydroxylation of alkyne	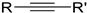	n*Cfu*CPO + H_2_O_2_ + X^−^	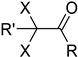	[67]
Halogenation and opening of cyclopropane	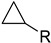	n*Cfu*CPO + H_2_O_2_ + X^−^	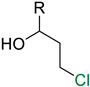	[67]
Halogenation of aromatic compound	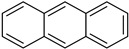	n*Cfu*CPO + H_2_O_2_ + Cl^−^	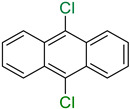	[68]
Halolactonization of unsaturated carboxylic acid	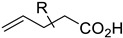	r*Ci*vCPO + H_2_O_2_ + X^−^	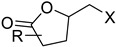	[69]
Haloetherification of alkenol	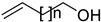	r*Ci*vCPO + H_2_O_2_ + X^−^	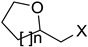	[69]

**Table 3:** Enzymatic halogenation catalyzed by some fungal flavin-dependent halogenases (r: recombinant protein).

Reaction	Substrate	Enzyme and Reagents	Product	Ref.
Halogenation of dihydro-resorcylide	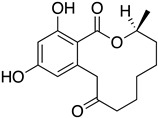	rRdc2 + O_2_ FADH_2_ + X^−^	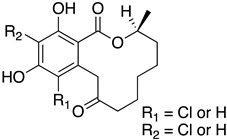	[70]
Halogenation of zearalenone	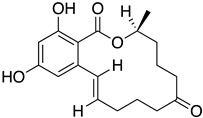	rRdc2 + O_2_ + FADH_2_ + X^−^	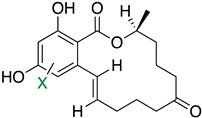	[70]
Halogenation of curvularin	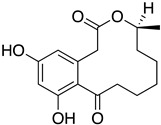	rRdc2 + O_2_ + FADH_2_ + X^−^	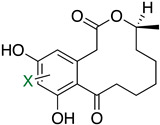	[70]
Halogenation of curcumin	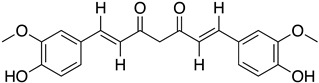	rRdc2 + O_2_ + FADH_2_ + X^−^	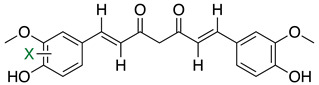	[70]
Chlorination of 4-hydroxyisoquinoline	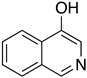	rRdc2 + O_2_ + FADH_2_ + Cl^−^	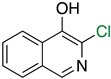	[71]
Chlorination of 6-hydroxyisoquinoline	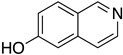	rRadH/rRdc2 + O_2_ + FADH_2_ + Cl^−^	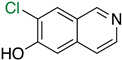	[71]

## Data Availability

Not applicable.

## Supplementary Materials

The following supporting information can be downloaded at: https://www.mdpi.com/article/10.3390/molecules27103157/s1, Figure S1: MCD assay: monochlorodimedon is halogenated to obtain dichlorodimedone if X^+^ = Cl^+^ or bromochlorodimedon if X^+^ = Br^+^. Figure S2: Alignment between vHPOs (fungi, algae and bacteria) and bacterial acid phosphatases PAP2. Figure S3: Summary of all putative sequences of fungal halogenation enzymes detected in both UniProt and MycoCosm databases. Distribution of all the detected sequences is found in each pie chart. The number of genomes for each phylogenetic branch available on MycoCosm database are given in grey under each fungal division. Number of sequences found in each database in January 2022 is indicated next to the caption in the summary part. All described enzymes are presented in the rectangle boxes in front of the corresponding fungal division.

## References

[1] Petty M.A. (1961). An Introduction to the Origin and Biochemistry of Microbial Halometabolites. Bacteriol. Rev..

[2] Zopf W. (1904). Zur Kenntniss Der Flechtenstoffe (12. Mittheilung.). Justus Liebigs Ann. Chem..

[3] Morris D.R., Hager L.P. (1966). Chloroperoxidase. I. Isolation and Properties of the Crystalline Glycoprotein. J. Biol. Chem..

[4] CRC Press Dictionary of Natural Products (DNP). https://dnp.chemnetbase.com/.

[5] Gribble G.W., Gribble G.W. (2010). Naturally Occuring Organohalogen Compounds—A Comprehensive Update.

[6] Dickson A.G., Goyet C., Dickson A.G., Goyet C. (1994). Handbook of Methods for the Analysis of the Various Parameters of the Carbon Dioxide System in Sea Water. Version 2. DOE.

[7] Van Santen J.A., Jacob G., Singh A.L., Aniebok V., Balunas M.J., Bunsko D., Neto F.C., Castaño-Espriu L., Chang C., Clark T.N. (2019). The Natural Products Atlas: An Open Access Knowledge Base for Microbial Natural Products Discovery. ACS Cent. Sci..

[8] Amend A., Burgaud G., Cunliffe M., Edgcomb V.P., Ettinger C.L., Gutiérrez M.H., Heitman J., Hom E.F.Y., Ianiri G., Jones A.C. (2019). Fungi in the Marine Environment: Open Questions and Unsolved Problems. mBio.

[9] Gladfelter A.S., James T.Y., Amend A.S. (2019). Marine Fungi. Curr. Biol. Mag..

[10] Roullier C., Guitton Y., Valery M., Amand S., Prado S., Robiou Du Pont T., Grovel O., Pouchus Y.F. (2016). Automated Detection of Natural Halogenated Compounds from LC-MS Profiles-Application to the Isolation of Bioactive Chlorinated Compounds from Marine-Derived Fungi. Anal. Chem..

[11] Atanasov A.G., Waltenberger B., Pferschy-Wenzig E.M., Linder T., Wawrosch C., Uhrin P., Temml V., Wang L., Schwaiger S., Heiss E.H. (2015). Discovery and Resupply of Pharmacologically Active Plant-Derived Natural Products: A Review. Biotechnol. Adv..

[12] Newman D.J., Cragg G.M. (2020). Natural Products as Sources of New Drugs over the Nearly Four Decades from 01/1981 to 09/2019. J. Nat. Prod..

[13] Jiang S., Zhang L., Cui D., Yao Z., Gao B., Lin J., Wei D. (2016). The Important Role of Halogen Bond in Substrate Selectivity of Enzymatic Catalysis. Sci. Rep..

[14] Julianti E., Abrian I.A., Wibowo M.S., Azhari M., Tsurayya N., Izzati F., Juanssilfero A.B., Bayu A., Rahmawati S.I., Putra M.Y. (2022). Secondary Metabolites from Marine-Derived Fungi and Actinobacteria as Potential Sources of Novel Colorectal Cancer Drugs. Mar. Drugs.

[15] Lu Y., Liu Y., Xu Z., Li H., Liu H., Zhu W. (2012). Halogen Bonding for Rational Drug Design and New Drug Discovery. Expert Opin. Drug Discov..

[16] Xu Z., Yang Z., Liu Y., Lu Y., Chen K., Zhu W. (2014). Halogen Bond: Its Role beyond Drug–Target Binding Affinity for Drug Discovery and Development. J. Chem. Inf. Model..

[17] Paul C., Pohnert G. (2011). Production and Role of Volatile Halogenated Compounds from Marine Algae. Nat. Prod. Rep..

[18] De Nys R., Steinberg P.D., Willemsen P., Dworjanyn S.A., Gabelish C.L., King R.J. (1995). Broad Spectrum Effects of Secondary Metabolites from the Red Alga Delisea Pulchra in Antifouling Assays. Biofouling.

[19] Ma X., Yu J., Jiang M., Wang M., Tang L., Wei M., Zhou Q. (2019). Mild and Regioselective Bromination of Phenols with TMSBr. Eur. J. Org. Chem..

[20] Tang R.-J., Milcent T., Crousse B. (2018). Regioselective Halogenation of Arenes and Heterocycles in Hexafluoroisopropanol. J. Org. Chem..

[21] Burgaud G., Edgcomb V., Hassett B.T., Kumar A., Li W., Mara P., Peng X., Philippe A., Phule P., Prado S., Stal L.J., Cretoiu M.S. (2022). Marine Fungi. The Marine Microbiome.

[22] Wang C., Lu H., Lan J., Zaman K., Cao S. (2021). A Review: Halogenated Compounds from Marine Fungi. Molecules.

[23] Grigoriev I.V., Nikitin R., Haridas S., Kuo A., Ohm R., Otillar R., Riley R., Salamov A., Zhao X., Korzeniewski F. (2014). MycoCosm Portal: Gearing up for 1000 Fungal Genomes. Nucleic Acids Res..

[24] Altschul S.F., Gish W., Miller W., Myers E.W., Lipman D.J. (1990). Basic Local Alignment Search Tool. J. Mol. Biol..

[25] Kumar S., Stecher G., Li M., Knyaz C., Tamura K. (2018). MEGA X: Molecular Evolutionary Genetics Analysis across Computing Platforms. Mol. Biol. Evol..

[26] Sehnal D., Rose A.S., Koča J., Burley S.K., Velankar S., Byska J., Krone M., Sommer B. (2018). Mol*: Towards a Common Library and Tools for Web Molecular Graphics. MolVA ’18, Proceedings of the Workshop on Molecular Graphics and Visual Analysis of Molecular Data.

[27] Fang G.-H., Kenigsberg P., Axley M.J., Nuell M., Hager L.P. (1986). Cloning and Sequencing of Chloroperoxidase CDNA. Nucleic Acids Res..

[28] Simons B.H., Barnett P., Vollenbroek E.G.M., Dekker H.L., Muijsers A.O., Messerschmidt A., Wever R. (1995). Primary Structure and Characterization of the Vanadium Chloroperoxidase from the Fungus Curvularia Inaequalis. Eur. J. Biochem..

[29] Barnett P., Hemrika W., Dekker H.L., Muijsers A.O., Renirie R., Wever R. (1998). Isolation, Characterization, and Primary Structure of the Vanadium Chloroperoxidase from the Fungus Embellisia Didymospora. J. Biol. Chem..

[30] Fraley A.E., Garcia-borràs M., Tripathi A., Khare D., Eduardo V., Tran H., Dan Q., Webb G.P., Watts K.R., Crews P. (2017). Function and Structure of MalA/MalA’, Iterative Halogenases for Late-Stage C–H Functionalization of Indole Alkaloids. J. Am. Chem. Soc..

[31] Reeves C.D., Hu Z., Reid R., Kealey J.T. (2008). Genes for the Biosynthesis of the Fungal Polyketides Hypothemycin from Hypomyces Subiculosus and Radicicol from Pochonia Chlamydosporia. Appl. Environ. Microbiol..

[32] Wang S., Xu Y., Maine E.A., Wijeratne E.M.K., Espinosa-Artiles P., Gunatilaka A.A.L., Molnár I. (2008). Functional Characterization of the Biosynthesis of Radicicol, an Hsp90 Inhibitor Resorcylic Acid Lactone from Chaetomium Chiversii. Chem. Biol..

[33] Ferrara M., Perrone G., Gambacorta L., Epifani F., Solfrizzo M., Gallo A. (2016). Identification of a Halogenase Involved in the Biosynthesis of Ochratoxin A in Aspergillus Carbonarius. Appl. Environ. Microbiol..

[34] Susca A., Proctor R.H., Morelli M., Haidukowski M., Gallo A., Logrieco A.F., Moretti A. (2016). Variation in Fumonisin and Ochratoxin Production Associated with Differences in Biosynthetic Gene Content in Aspergillus Niger and A. Welwitschiae Isolates from Multiple Crop and Geographic Origins. Front. Microbiol..

[35] Xu X., Liu L., Zhang F., Wang W., Li J., Guo L., Che Y., Liu G. (2014). Identification of the First Diphenyl Ether Gene Cluster for Pestheic Acid Biosynthesis in Plant Endophyte Pestalotiopsis Fici. ChemBioChem.

[36] Nielsen M.T., Nielsen J.B., Anyaogu D.C., Holm D.K., Nielsen K.F., Larsen T.O., Mortensen U.H. (2013). Heterologous Reconstitution of the Intact Geodin Gene Cluster in Aspergillus Nidulans through a Simple and Versatile PCR Based Approach. PLoS ONE.

[37] Cacho R.A., Chooi Y.H., Zhou H., Tang Y. (2013). Complexity Generation in Fungal Polyketide Biosynthesis: A Spirocycle-Forming P450 in the Concise Pathway to the Antifungal Drug Griseofulvin. ACS Chem. Biol..

[38] Chankhamjon P., Boettger-Schmidt D., Scherlach K., Urbansky B., Lackner G., Kalb D., Dahse H.M., Hoffmeister D., Hertweck C. (2014). Biosynthesis of the Halogenated Mycotoxin Aspirochlorine in Koji Mold Involves a Cryptic Amino Acid Conversion. Angew. Chem.-Int. Ed..

[39] Araki Y., Awakawa T., Matsuzaki M., Cho R., Matsuda Y., Hoshino S., Shinohara Y., Yamamoto M., Kido Y., Inaoka D.K. (2019). Complete Biosynthetic Pathways of Ascofuranone and Ascochlorin in Acremonium Egyptiacum. Proc. Natl. Acad. Sci. USA.

[40] Wick J., Heine D., Lackner G., Misiek M., Tauber J., Jagusch H., Hertweck C., Hoffmeister D. (2016). A Fivefold Parallelized Biosynthetic Process Secures Chlorination of Armillaria Mellea (Honey Mushroom) Toxins. Appl. Environ. Microbiol..

[41] Ugai T., Minami A., Tanaka S., Ozaki T., Liu C., Shigemori H., Hashimoto M., Oikawa H. (2020). Biosynthetic Machinery of 6-Hydroxymellein Derivatives Leading to Cyclohelminthols and Palmaenones. ChemBioChem.

[42] Winter J.M., Sato M., Sugimoto S., Chiou G., Garg N.K., Tang Y., Watanabe K. (2012). Identification and Characterization of the Chaetoviridin and Chaetomugilin Gene Cluster in Chaetomium Globosum Reveal Dual Functions of an Iterative Highly-Reducing Polyketide Synthase. J. Am. Chem. Soc..

[43] Axley M.J., Kenigsberg P., Hager L.P. (1986). Fructose Induces and Glucose Represses Chloroperoxidase MRNA Levels. J. Biol. Chem..

[44] Blin K., Shaw S., Kloosterman A.M., Charlop-Powers Z., van Wezel G.P., Medema M.H., Weber T. (2021). AntiSMASH 6.0: Improving Cluster Detection and Comparison Capabilities. Nucleic Acids Res..

[45] Menon B.R.K., Richmond D., Menon N. (2020). Halogenases for Biosynthetic Pathway Engineering: Toward New Routes to Naturals and Non-Naturals. Catal. Rev..

[46] Crowe C., Molyneux S., Sharma S.V., Zhang Y., Gkotsi D.S., Connaris H., Goss R.J.M. (2021). Halogenases: A Palette of Emerging Opportunities for Synthetic Biology–Synthetic Chemistry and C–H Functionalisation. Chem. Soc. Rev..

[47] Dachwitz S., Widmann C., Frese M., Niemann H.H., Sewald N. (2020). Enzymatic Halogenation: Enzyme Mining, Mechanisms, and Implementation in Reaction Cascades. Amino Acids, Peptides and Proteins.

[48] Hager L., Morris D., Brown F., Eberwein H. (1966). Chloroperoxidase: Utilization of Halogen Anions. J. Biol. Chem..

[49] Verhaeghe E., Buisson D., Zekri E., Leblanc C., Potin P., Ambroise Y. (2008). A Colorimetric Assay for Steady-State Analyses of Iodo- and Bromoperoxidase Activities. Anal. Biochem..

[50] Jordan P., Vilter H. (1990). Native Bromoperoxidases Do Not Bind to Nitrocellulose: Use of DEAE-Cellulose as an Alternative in Blotting. Electrophoresis.

[51] Nag N., Khan H., Tripathi T. (2022). Strategies to Improve the Expression and Solubility of Recombinant Proteins in *E. Coli*. Advances in Protein Molecular and Structural Biology Methods.

[52] Zemella A., Thoring L., Hoffmeister C., Kubick S. (2015). Cell-Free Protein Synthesis: Pros and Cons of Prokaryotic and Eukaryotic Systems. ChemBioChem.

[53] Mamipour M., Yousefi M., Hasanzadeh M. (2017). An Overview on Molecular Chaperones Enhancing Solubility of Expressed Recombinant Proteins with Correct Folding. Int. J. Biol. Macromol..

[54] Hemrika W., Renirie R., Macedo-Ribeiro S., Messerschmidt A., Wever R. (1999). Heterologous Expression of the Vanadium-Containing Chloroperoxidase from Curvularia Inaequalis in Saccharomyces Cerevisiae and Site-Directed Mutagenesis of the Active Site Residues His496, Lys353, Arg360, and Arg490. J. Biol. Chem..

[55] Conesa A., van de Velde F., van Rantwijk F., Sheldon R.A., van den Hondel C.A.M.J.J., Punt P.J. (2001). Expression of the Caldariomyces Fumago Chloroperoxidase in Aspergillus Niger and Characterization of the Recombinant Enzyme. J. Biol. Chem..

[56] Zhou H., Qiao K., Gao Z., Vederas J.C., Tang Y. (2010). Insights into Radicicol Biosynthesis via Heterologous Synthesis of Intermediates and Analogs. J. Biol. Chem..

[57] Messerschmidt A., Prade L., Wever R. (1997). Implications for the Catalytic Mechanism of the Vanadium-Containing Enzyme Chloroperoxidase from the Fungus Curvularia Inaequalis by X-Ray Structures of the Native and Peroxide Form. Biol. Chem..

[58] Pickard M.A., Kadima T.A., Carmichael R.D. (1991). Chloroperoxidase, a Peroxidase with Potential. J. Ind. Microbiol..

[59] Bormann S., Gomez Baraibar A., Ni Y., Holtmann D., Hollmann F. (2015). Specific Oxyfunctionalisations Catalysed by Peroxygenases: Opportunities, Challenges and Solutions. Catal. Sci. Technol..

[60] Aoun S., Baboulène M. (1998). Regioselective Bromohydroxylation of Alkenes Catalyzed by Chloroperoxidase: Advantages of the Immobilization of Enzyme on Talc. J. Mol. Catal.-B Enzym..

[61] Wischang D., Hartung J. (2012). Bromination of Phenols in Bromoperoxidase-Catalyzed Oxidations. Tetrahedron.

[62] van Deurzen M.P.J., van Rantwijk F., Sheldon R.A. (1996). Synthesis of Substituted Oxindoles by Chloroperoxidase Catalyzed Oxidation of Indoles. J. Mol. Catal. B: Enzym..

[63] Höfler G.T., But A., Hollmann F. (2019). Haloperoxidases as Catalysts in Organic Synthesis. Org. Biomol. Chem..

[64] Fernández-Fueyo E., H Younes S.H., van Rootselaar S., Aben W.M., Renirie R., Wever R., Holtmann D., J T Rutjes F.P., Hollmann F. (2016). A Biocatalytic Aza-Achmatowicz Reaction. ACS Catal..

[65] Yaipakdee P., Robertson L.W. (2001). Enzymatic Halogenation of Flavanones and Flavones. Phytochemistry.

[66] Franssen M.C.R., van der Plas H.C. (1987). The Chlorination of Barbituric Acid and Some of Its Derivatives by Chloroperoxidase. Bioorg. Chem..

[67] Geigert J., Neidleman S.L., Dalietos D.J. (1983). Novel Haloperoxidase Substrates. Alkynes and Cyclopropanes. J. Biol. Chem..

[68] Vázquez-Duhalt R., Ayala M., Márquez-Rocha F.J. (2001). Biocatalytic Chlorination of Aromatic Hydrocarbons by Chloroperoxidase of Caldariomyces Fumago. Phytochemistry.

[69] Younes S.H.H., Tieves F., Lan D., Wang Y., Süss P., Brundiek H., Wever R., Hollmann F. (2019). Chemoenzymatic Halocyclization of γ,δ-Unsaturated Carboxylic Acids and Alcohols. ChemSusChem.

[70] Zeng J., Zhan J. (2010). A Novel Fungal Flavin-Dependent Halogenase for Natural Product Biosynthesis. ChemBioChem.

[71] Zeng J., Lytle A.K., Gage D., Johnson S.J., Zhan J. (2013). Specific Chlorination of Isoquinolines by a Fungal Flavin-Dependent Halogenase. Bioorg. Med. Chem. Lett..

[72] Timmins A., De Visser S.P. (2015). Enzymatic Halogenases and Haloperoxidases: Computational Studies on Mechanism and Function. Adv. Protein Chem. Struct. Biol..

[73] Sundaramoorthy M., Terner J., Poulos T.L. (1995). The Crystal Structure of Chloroperoxidase: A Heme Peroxidase-Cytochrome P450 Functional Hybrid. Structure.

[74] Zong Q., Osmulski P.A., Hager L.P. (1995). High-Pressure-Assisted Reconstitution of Recombinant Chloroperoxidase. Biochemistry.

[75] Butler A., Sandy M. (2009). Mechanistic Considerations of Halogenating Enzymes. Nature.

[76] Kühnel K., Blankenfeldt W., Terner J., Schlichting I. (2006). Crystal Structures of Chloroperoxidase with Its Bound Substrates and Complexed with Formate, Acetate, and Nitrate. J. Biol. Chem..

[77] Sundaramoorthy M., Terner J., Poulos T.L. (1998). Stereochemistry of the Chloroperoxidase Active Site: Crystallographic and Molecular-Modeling Studies. Chem. Biol..

[78] Shaik S., Kumar D., De Visser S.P. (2008). A Valence Bond Modeling of Trends in Hydrogen Abstraction Barriers and Transition States of Hydroxylation Reactions Catalyzed by Cytochrome P450 Enzymes. J. Am. Chem. Soc..

[79] Hofrichter M., Ullrich R. (2006). Heme-Thiolate Haloperoxidases: Versatile Biocatalysts with Biotechnological and Environmental Significance. Appl. Microbiol. Biotechnol..

[80] Farhangrazi Z.S., Sinclair R., Yamazaki I., Powers L.S. (1992). Haloperoxidase Activity of Phanerochaete Chrysosporium Lignin Peroxidases H2 and H8. Biochemistry.

[81] Ullrich R., Nueske J., Scheibner K., Spantzel J., Hofrichter M. (2004). Novel Haloperoxidase from the Agaric Basidiomycete. Appl. Environ. Microbiol..

[82] Piontek K., Ullrich R., Liers C., Diederichs K., Plattner D.A., Hofrichter M. (2010). Crystallization of a 45 KDa Peroxygenase/Peroxidase from the Mushroom Agrocybe Aegerita and Structure Determination by SAD Utilizing Only the Haem Iron. Acta Crystallogr. Sect. F: Struct. Biol. Cryst. Commun..

[83] Zamocky M., Obinger C., Torres E., Ayala M. (2010). Biocatalysis Based on Heme Peroxidases.

[84] China H., Okada Y., Dohi T. (2015). The Multiple Reactions in the Monochlorodimedone Assay: Discovery of Unique Dehalolactonizations under Mild Conditions. Asian J. Org. Chem..

[85] Conesa A., Punt P.J., Van Den Hondel C.A.M.J.J. (2002). Fungal Peroxidases: Molecular Aspects and Applications. J. Biotechnol..

[86] Cherry J.R., Lamsa M.H., Schneider P., Vind J., Svendsen A., Jones A., Pedersen A.H. (1999). Directed Evolution of a Fungal Peroxidase. Nat. Biotechnol..

[87] Vilter H. (1984). Peroxidases from Phaeophyceae: A Vanadium(V)-Dependent Peroxidase from Ascophyllum Nodosum. Phytochemistry.

[88] Agarwal V., Miles Z.D., Winter J.M., Eustáquio A.S., el Gamal A.A., Moore B.S. (2017). Enzymatic Halogenation and Dehalogenation Reactions: Pervasive and Mechanistically Diverse. Chem. Rev..

[89] Bertini I., Sigel A., Sigel H., Bertini I., Sigel A., Sigel H. (2006). Handbook of Metalloproteins.

[90] Leblanc C., Vilter H., Fournier J.B., Delage L., Potin P., Rebuffet E., Michel G., Solari P.L., Feiters M.C., Czjzek M. (2015). Vanadium Haloperoxidases: From the Discovery 30 Years Ago to X-ray Crystallographic and V K-Edge Absorption Spectroscopic Studies. Coord. Chem. Rev..

[91] Messerschmidt A., Wever R. (1996). X-ray Structure of a Vanadium-Containing Enzyme: Chloroperoxidase from the Fungus Curvularia Inaequalis. Proc. Natl. Acad. Sci. USA.

[92] Fournier J.-B., Leblanc C., La Barre S., Kornprobst J.-M. (2014). Halogenation and Vanadium Haloperoxidases. Outstanding Marine Molecules.

[93] Neuwald A.F. (1997). An Unexpected Structural Relationship between Integral Membrane Phosphatases and Soluble Haloperoxidases. Protein Sci..

[94] Renirie R., Hemrika W., Wever R. (2000). Peroxidase and Phosphatase Activity of Active-Site Mutants of Vanadium Chloroperoxidase from the Fungus Curvularia Inaequalis. Implications for the Catalytic Mechanisms. J. Biol. Chem..

[95] Hasan Z., Renirie R., Kerkman R., Ruijssenaars H.J., Hartog A.F., Wever R. (2006). Laboratory-Evolved Vanadium Chloroperoxidase Exhibits 100-Fold Higher Halogenating Activity at Alkaline PH: Catalytic Effects from First and Second Coordination Sphere Mutations. J. Biol. Chem..

[96] Fournier J.B., Rebuffet E., Delage L., Grijol R., Meslet-Cladière L., Rzonca J., Potin P., Michel G., Czjzek M., Leblanc C. (2014). The Vanadium Iodoperoxidase from the Marine Flavobacteriaceae Species Zobellia Galactanivorans Reveals Novel Molecular and Evolutionary Features of Halide Specificity in the Vanadium Haloperoxidase Enzyme Family. Appl. Environ. Microbiol..

[97] Butler A., Carter-Franklin J.N. (2004). The Role of Vanadium Bromoperoxidase in the Biosynthesis of Halogenated Marine Natural Products. Nat. Prod. Rep..

[98] Carter-Franklin J.N., Butler A. (2004). Vanadium Bromoperoxidase-Catalyzed Biosynthesis of Halogenated Marine Natural Products. J. Am. Chem. Soc..

[99] Bar-Nun N., Shcolnick S., Mayer A.M. (2002). Presence of a Vanadium-Dependent Haloperoxidase in Botrytis Cinerea. FEMS Microbiol. Lett..

[100] Plat H., Krenn B.E., Wever R. (1987). The Bromoperoxidase from the Lichen Xanthoria Parietina Is a Novel Vanadium Enzyme. Biochem. J..

[101] Chen Z. (2022). Recent Development of Biomimetic Halogenation Inspired by Vanadium Dependent Haloperoxidase. Coord. Chem. Rev..

[102] Dong J.J., Ferná Ndez-Fueyo E., Li J., Guo Z., Renirie R., Wever R., Hollmann F. (2017). Halofunctionalization of Alkenes by Vanadium Chloroperoxidase from Curvularia Inaequalis. Chem. Commun..

[103] Naapuri J.M., Wagner P.K., Hollmann F., Deska J. (2022). Enzymatic Bromocyclization of A- and Γ-Allenols by Chloroperoxidase from *Curvularia inaequalis*. ChemistryOpen.

[104] Fernández-Fueyo E., van Wingerden M., Renirie R., Wever R., Ni Y., Holtmann D., Hollmann F. (2015). Chemoenzymatic Halogenation of Phenols by Using the Haloperoxidase from Curvularia Inaequalis. ChemCatChem.

[105] Wiesner W., van Pée K.H., Lingens F. (1988). Purification and Characterization of a Novel Bacterial Non-Heme Chloroperoxidase from Pseudomonas Pyrrocinia. J. Biol. Chem..

[106] Hofmann B., Tölzer S., Pelletier I., Altenbuchner J., van Pée K.H., Hecht H.J. (1998). Structural Investigation of the Cofactor-Free Chloroperoxidases. J. Mol. Biol..

[107] Pelletier I., Altenbuchner J., Mattes R. (1995). A Catalytic Triad Is Required by the Non-Heme Haloperoxidases to Perform Halogenation. Biochim. Biophys. Acta (BBA)/Protein Struct. Mol..

[108] Picard M., Gross J., Lübbert E., Tölzer S., Krauss S., Van Pée K.H., Berkessel A. (1997). Metal-Free Bacterial Haloperoxidases as Unusual Hydrolases: Activation of H_2_O_2_ by the Formation of Peracetic Acid. Angew. Chem. Int. Ed. Engl..

[109] Xu G., Wang B.G. (2016). Independent Evolution of Six Families of Halogenating Enzymes. PLoS ONE.

[110] Hecht H.J., Sobek H., Haag T., Pfeifer O., van Pée K.-H. (1994). The Metal-Ion-Free Oxidoreductase from Streptomyces Aureofaciens Has an α/β Hydrolase Fold. Nat. Struct. Biol..

[111] Dairi T., Nakano T., Aisaka K., Katsumata R., Hasegawa M. (1995). Cloning and Nucleotide Sequence of the Gene Responsible for Chlorination of Tetracycline. Biosci. Biotechnol. Biochem..

[112] Neubauer P.R., Widmann C., Wibberg D., Schröder L., Frese M., Kottke T., Kalinowski J., Niemann H.H., Sewald N. (2018). A Flavin-Dependent Halogenase from Metagenomic Analysis Prefers Bromination over Chlorination. PLoS ONE.

[113] Latham J., Brandenburger E., Shepherd S.A., Menon B.R.K., Micklefield J. (2018). Development of Halogenase Enzymes for Use in Synthesis. Chem. Rev..

[114] Dong C., Flecks S., Unversucht S., Haupt C., Naismith J.H. (2005). The Structure of Tryptophan 7-Halogenase (PrnA) Suggests a Mechanism for Regioselective Chlorination. Science.

[115] Yeh E., Blasiak L.C., Koglin A., Drennan C.L., Walsh C.T. (2007). Chlorination by a Long-Lived Intermediate in the Mechanism of Flavin-Dependent Halogenases. Biochemistry.

[116] Flecks S., Patallo E.P., Zhu X., Ernyei A.J., Seifert G. (2008). New Insights into the Mechanism of Enzymatic Chlorination of Tryptophan. Angew. Chem. Int. Ed. Engl..

[117] Miller K.A., Figueroa M., Valente M.W.N., Greshock T.J., Mata R., Williams R.M. (2008). Calmodulin Inhibitory Activity of the Malbrancheamides and Various Analogs. Bioorg. Med. Chem. Lett..

[118] Andorfer C., Lewis J. (2018). Understanding and Improving the Activity of Flavin Dependent Halogenases via Random and Targeted Mutagenesis. Annu. Rev. Biochem..

[119] Eichhorn E., Van Der Ploeg J.R., Leisinger T. (1999). Characterization of a Two-Component Alkanesulfonate Monooxygenase from Escherichia Coli. J. Biol. Chem..

[120] Yeh E., Cole L.J., Barr E.W., Bollinger J.M., Ballou D.P., Walsh C.T. (2006). Flavin Redox Chemistry Precedes Substrate Chlorination during the Reaction of the Flavin-Dependent Halogenase RebH. Biochemistry.

[121] Tran P.N., Yen M.R., Chiang C.Y., Lin H.C., Chen P.Y. (2019). Detecting and Prioritizing Biosynthetic Gene Clusters for Bioactive Compounds in Bacteria and Fungi. Appl. Microbiol. Biotechnol..

[122] Walsh C.T., Garneau-Tsodikova S., Howard-Jones A.R. (2006). Biological Formation of Pyrroles: Nature’s Logic and Enzymatic Machinery. Nat. Prod. Rep..

[123] Hohaus K., Altmann A., Burd W., Fischer I., Hammer P.E., Hill D.S., Ligon J.M., van Pée K.-H. (1997). NADH-Dependent Halogenases Are More Likely To Be Involved in Halometabolite Biosynthesis Than Haloperoxidases. Angew. Chem. Int. Ed. Engl..

[124] Tola M., Kebede B. (2016). Occurrence, Importance and Control of Mycotoxins: A Review. Cogent Food Agric..

[125] Shinohara C., Chikanishi T., Nakashima S., Hashimoto A., Hamanaka A., Endo A., Hasumi K. (2000). Enhancement of Fibrinolytic Activity of Vascular Endothelial Cells by Chaetoglobosin A, Crinipellin B, Geodin and Triticone B. J. Antibiot..

[126] Chooi Y.-H., Cacho R., Tang Y. (2010). Identification of the Viridicatumtoxin and Griseofulvin Gene Clusters from Penicillium Aethiopicum. Chem. Biol..

[127] Chankhamjon P., Tsunematsu Y., Ishida-Ito M., Sasa Y., Meyer F., Boettger-Schmidt D., Urbansky B., Menzel K.D., Scherlach K., Watanabe K. (2016). Regioselective Dichlorination of a Non-Activated Aliphatic Carbon Atom and Phenolic Bismethylation by a Multifunctional Fungal Flavoenzyme. Angew. Chem.-Int. Ed..

[128] Gkotsi D.S., Dhaliwal J., McLachlan M.M., Mulholand K.R., Goss R.J. (2018). Halogenases: Powerful Tools for Biocatalysis (Mechanisms Applications and Scope). Curr. Opin. Chem. Biol..

[129] Frese M., Sewald N. (2015). Enzymatic Halogenation of Tryptophan on a Gram Scale. Angew. Chem.-Int. Ed..

[130] Büchler J., Papadopoulou A., Buller R. (2019). Recent Advances in Flavin-Dependent Halogenase Biocatalysis: Sourcing, Engineering, and Application. Catalysts.

[131] Schnepel C., Minges H., Frese M., Sewald N. (2016). A High-Throughput Fluorescence Assay to Determine the Activity of Tryptophan Halogenases. Angew. Chem.-Int. Ed..

[132] Payne J.T., Poor C.B., Lewis J.C. (2015). Directed Evolution of Rebh for Site-Selective Halogenation of Large Biologically Active Molecules. Angew. Chem.-Int. Ed..

[133] Roy A.D., Grüschow S., Cairns N., Goss R.J.M. (2010). Gene Expression Enabling Synthetic Diversification of Natural Products: Chemogenetic Generation of Pacidamycin Analogs. J. Am. Chem. Soc..

[134] Glenn W.S., Nims E., O’Connor S.E. (2011). Reengineering a Tryptophan Halogenase to Preferentially Chlorinate a Direct Alkaloid Precursor. J. Am. Chem. Soc..

[135] Fraley A.E., Sherman D.H. (2020). Enzyme Evolution in Fungal Indole Alkaloid Biosynthesis. FEBS J..

[136] Hillwig M.L., Liu X. (2014). A New Family of Iron-Dependent Halogenases Acts on Freestanding Substrates. Nat. Chem. Biol..

[137] Matthews M., Krest C., Barr E., Vaillancourt F., Walsh C., Green M., Krebs C., Bollinger J. (2009). Substrate-Triggered Formation and Remarkable Stability of the CH-Cleaving Chloroferryl Intermediate in the Aliphatic Halogenase, SyrB2. Biochemistry.

[138] Zhang X., Wang Z., Gao J., Liu W. (2020). Chlorination versus Hydroxylation Selectivity Mediated by the Non-Heme Iron Halogenase WelO5. Phys. Chem. Chem. Phys..

[139] Vaillancourt F.H., Yin J., Walsh C.T. (2005). From The Cover: SyrB2 in Syringomycin E Biosynthesis Is a Nonheme FeII -Ketoglutarate- and O2-Dependent Halogenase. Proc. Natl. Acad. Sci. USA.

[140] Jiang W., Heemstra J.R., Forseth R.R., Neumann C.S., Manaviazar S., Schroeder F.C., Hale K.J., Walsh C.T. (2011). Biosynthetic Chlorination of the Piperazate Residue in Kutzneride Biosynthesis by KthP. Biochemistry.

[141] Rhew R.C., Østergaard L., Saltzman E.S., Yanofsky M.F. (2003). Genetic Control of Methyl Halide Production in Arabidopsis. Curr. Biol..

[142] Watling R., Harper D.B. (1998). Chloromethane Production by Wood-Rotting Fungi and an Estimate of the Global Flux to the Atmosphere. Mycol. Res..

[143] McKinnie S.M.K., Miles Z.D., Jordan P.A., Awakawa T., Pepper H.P., Murray L.A.M., George J.H., Moore B.S. (2018). Total Enzyme Syntheses of Napyradiomycins A1 and B1. J. Am. Chem. Soc..

[144] Kaysser L., Bernhardt P., Nam S.-J., Loesgen S., Ruby J.G., Skewes-Cox P., Jensen P.R., Fenical W., Moore B.S. (2012). Merochlorins A–D, Cyclic Meroterpenoid Antibiotics Biosynthesized in Divergent Pathways with Vanadium-Dependent Chloroperoxidases. J. Am. Chem. Soc..

[145] Murphy C.D., Schaffrath C., O’Hagan D. (2003). Fluorinated Natural Products: The Biosynthesis of Fluoroacetate and 4-Fluorothreonine in Streptomyces Cattleya. Chemosphere.

[146] Sander T., Freyss J., Von Korff M., Rufener C. (2015). DataWarrior: An Open-Source Program for Chemistry Aware Data Visualization and Analysis. J. Chem. Inf. Model..

[147] Djoumbou Feunang Y., Eisner R., Knox C., Chepelev L., Hastings J., Owen G., Fahy E., Steinbeck C., Subramanian S., Bolton E. (2016). ClassyFire: Automated Chemical Classification with a Comprehensive, Computable Taxonomy. J. Cheminform..

[148] Arai M., Yamamoto K., Namatame I., Tomoda H., Omura S. (2003). New Monordens Produced by Amidepsine-Producing Fungus Humicola Sp. FO-2942. J. Antibiot..

[149] Shigemori H., Tanabe Y., Matsumoto T., Hosoya T., Sato H. (2013). Palmaerins A-D, New Chlorinated and Brominated Dihydroisocoumarins with Antimicrobial and Plant Growth Regulating Activities from Discomycete Lachnum Palmae. HETEROCYCLES.

[150] Li H., Liao Z.-B., Tang D., Han W.-B., Zhang Q., Gao J.-M. (2018). Polyketides from Two Chaetomium Species and Their Biological Functions. J. Antibiot..

[151] Krohn K., Bahramsari R., Flörke U., Ludewig K., Kliche-Spory C., Michel A., Aust H.-J., Draeger S., Schulz B., Antus S. (1997). Dihydroisocoumarins from Fungi: Isolation, Structure Elucidation, Circular Dichroism and Biological Activity. Phytochemistry.

[152] Ogawa T., Ando K., Aotani Y., Shinoda K., Tanaka T., Tsukuda E., Yoshida M., Matsuda Y. (1995). RES-1214-1 and-2, Novel Non-Peptidic Endothelin Type A Receptor Antagonists Produced by *Pestalotiopsis* Sp.. J. Antibiot..

[153] Millot M., Tomasi S., Studzinska E., Rouaud I., Boustie J. (2009). Cytotoxic Constituents of the Lichen Diploicia Canescens. J. Nat. Prod..

[154] Wang J.-F., Zhou L.-M., Chen S.-T., Yang B., Liao S.-R., Kong F.-D., Lin X.-P., Wang F.-Z., Zhou X.-F., Liu Y.-H. (2018). New Chlorinated Diphenyl Ethers and Xanthones from a Deep-Sea-Derived Fungus Penicillium Chrysogenum SCSIO 41001. Fitoterapia.

[155] Rukachaisirikul V., Satpradit S., Klaiklay S., Phongpaichit S., Borwornwiriyapan K., Sakayaroj J. (2014). Polyketide Anthraquinone, Diphenyl Ether, and Xanthone Derivatives from the Soil Fungus Penicillium Sp. PSU-RSPG99. Tetrahedron.

[156] Bunbamrung N., Intaraudom C., Dramae A., Boonyuen N., Chanthaket R., Rachtawee P., Pittayakhajonwut P. (2018). Antagonistic Metabolites Produced by the Fungus Curvularia Sp. BCC52426 against Aspergillus Sp. BCC51998. Phytochem. Lett..

[157] Yao Q., Wang J., Zhang X., Nong X., Xu X., Qi S. (2014). Cytotoxic Polyketides from the Deep-Sea-Derived Fungus Engyodontium Album DFFSCS021. Mar. Drugs.

[158] Niu S., Liu D., Hu X., Proksch P., Shao Z., Lin W. (2014). Spiromastixones A–O, Antibacterial Chlorodepsidones from a Deep-Sea-Derived *Spiromastix* Sp. Fungus. J. Nat. Prod..

[159] Morshed M.T., Vuong D., Crombie A., Lacey A.E., Karuso P., Lacey E., Piggott A.M. (2018). Expanding Antibiotic Chemical Space around the Nidulin Pharmacophore. Org. Biomol. Chem..

[160] Chen W., Chen R., Liu Q., He Y., He K., Ding X., Kang L., Guo X., Xie N., Zhou Y. (2017). Orange, Red, Yellow: Biosynthesis of Azaphilone Pigments in Monascus Fungi. Chem. Sci..

[161] Sato M., Winter J.M., Kishimoto S., Noguchi H., Tang Y., Watanabe K. (2016). Combinatorial Generation of Chemical Diversity by Redox Enzymes in Chaetoviridin Biosynthesis. Org. Lett..

[162] Chen C., Tao H., Chen W., Yang B., Zhou X., Luo X., Liu Y. (2020). Recent Advances in the Chemistry and Biology of Azaphilones. RSC Adv..

[163] McMurry J., Brooks C. (2012). Organic Chemistry.

[164] Kim K.M., Park I.H. (2004). A Convenient Halogenation of α,β-Unsaturated Carbonyl Compounds with OXONE^®^ and Hydrohalic Acid (HBr, HCl). Synthesis.

[165] Mukhopadhyay T., Bhat R.G., Roy K., Vijayakumar E.K.S., Ganguli B.N. (1998). Aranochlor A and Aranochlor B, Two New Metabolites from Pseudoarachniotus Roseus: Production, Isolation, Structure Elucidation and Biological Properties. J. Antibiot..

[166] Amagata T., Tanaka M., Yamada T., Minoura K., Numata A. (2008). Gymnastatins and Dankastatins, Growth Inhibitory Metabolites of a Gymnascella Species from a Halichondria Sponge. J. Nat. Prod..

[167] Konaklieva M.I., Dahl M.L., Turos E. (1992). Halogenation Reactions of Epoxides. Tetrahedron Lett..

[168] Engler M., Anke T., Sterner O., Brandt U. (1997). Pterulinic Acid and Pterulone, Two Novel Inhibitors of NADH: Ubiquinone Oxidoreductase (Complex I) Produced by a Pterula Species. I. Production, Isolation and Biological Activities. J. Antibiot..

[169] Kornsakulkarn J., Thongpanchang C., Chainoy R., Choowong W., Nithithanasilp S., Thongpanchang T. (2010). Bioactive Metabolites from Cultures of Basidiomycete Favolaschia Tonkinensis. J. Nat. Prod..

[170] Lemaire P., Balme G., Desbordes P., Vors J.P. (2003). Efficient Syntheses of Pterulone, Pterulone B and Related Analogues. Org. Biomol. Chem..

[171] Almeida C., Pérez-Victoria I., González-Menéndez V., de Pedro N., Martín J., Crespo G., Mackenzie T., Cautain B., Reyes F., Vicente F. (2018). Non-Geminal Aliphatic Dihalogenation Pattern in Dichlorinated Diaporthins from Hamigera Fusca NRRL 35721. J. Nat. Prod..

[172] Cai R., Wu Y., Chen S., Cui H., Liu Z., Li C., She Z. (2018). Peniisocoumarins A–J: Isocoumarins from Penicillium Commune QQF-3, an Endophytic Fungus of the Mangrove Plant Kandelia Candel. J. Nat. Prod..

[173] Bonini C., Righi G. (1994). Regio- and Chemoselective Synthesis of Halohydrins by Cleavage of Oxiranes with Metal Halides. Synthesis.

[174] Stewart C.A., VanderWerf C.A. (1954). Reaction of Propylene Oxide with Hydrogen Halides. J. Am. Chem. Soc..

[175] Schafhauser T., Kirchner N., Kulik A., Huijbers M.M.E., Flor L., Caradec T., Fewer D.P., Gross H., Jacques P., Jahn L. (2016). The Cyclochlorotine Mycotoxin Is Produced by the Nonribosomal Peptide Synthetase CctN in Talaromyces Islandicus (‘Penicillium Islandicum’). Environ. Microbiol..

[176] Jiang Y., Ozaki T., Liu C., Igarashi Y., Ye Y., Tang S., Ye T., Maruyama J., Minami A., Oikawa H. (2021). Biosynthesis of Cyclochlorotine: Identification of the Genes Involved in Oxidative Transformations and Intramolecular O,N-Transacylation. Org. Lett..

[177] Kropp P.J., Daus K.A., Crawford S.D., Tubergen M.W., Kepler K.D., Craig S.L., Wilson V.P. (1990). Surface-Mediated Reactions. 1. Hydrohalogenation of Alkenes and Alkynes. J. Am. Chem. Soc..

[178] Wang W.-X., Cheng G.-G., Li Z.-H., Ai H.-L., He J., Li J., Feng T., Liu J.-K. (2019). Curtachalasins, Immunosuppressive Agents from the Endophytic Fungus Xylaria Cf. Curta. Org. Biomol. Chem..

[179] Wang W.-X., Lei X., Yang Y.-L., Li Z.-H., Ai H.-L., Li J., Feng T., Liu J.-K. (2019). Xylarichalasin A, a Halogenated Hexacyclic Cytochalasan from the Fungus Xylaria Cf. Curta. Org. Lett..

[180] Hemberger Y., Xu J., Wray V., Proksch P., Wu J., Bringmann G. (2013). Pestalotiopens A and B: Stereochemically Challenging Flexible Sesquiterpene-Cyclopaldic Acid Hybrids from *Pestalotiopsis* Sp.. Chem.-A Eur. J..

[181] Wolpert T.J., Macko V., Acklin W., Jaun B., Arigoni D. (1986). Structure of Minor Host-Selective Toxins From *Cochliobolus victoriae*. Experientia.

[182] Ngokpol S., Suwakulsiri W., Sureram S., Lirdprapamongkol K., Aree T., Wiyakrutta S., Mahidol C., Ruchirawat S., Kittakoop P. (2015). Drimane Sesquiterpene-Conjugated Amino Acids from a Marine Isolate of the Fungus *Talaromyces minioluteus* (*Penicillium minioluteum*). Mar. Drugs.

[183] Kessler S.C., Zhang X., McDonald M.C., Gilchrist C.L.M., Lin Z., Rightmyer A., Solomon P.S., Turgeon B.G., Chooi Y.-H. (2020). Victorin, the Host-Selective Cyclic Peptide Toxin from the Oat Pathogen Cochliobolus Victoriae, Is Ribosomally Encoded. Proc. Natl. Acad. Sci. USA.

[184] Flatt P.M., O’Connell S.J., McPhail K.L., Zeller G., Willis C.L., Sherman D.H., Gerwick W.H. (2006). Characterization of the Initial Enzymatic Steps of Barbamide Biosynthesis. J. Nat. Prod..

