# Supplementary material for: Halogenation in Fungi: What Do We Know and What Remains to Be Discovered?
Source: Molecules. 2022 May 14;27(10):3157. doi: 10.3390/molecules27103157 (PMC9144378; doi:10.3390/molecules27103157)
Supplement: Supplementary file 1 [file molecules-27-03157-s001.zip › molecules-1696495-supplementary.pdf]

Supplementary Material for:

Halogenation in Fungi: What do We Know and What Remains to Be Discovered?

Bastien Cochereau[1,2], Laurence Meslet-Cladière[2], Yves-François Pouchus[1], Olivier Grovel[1] and Catherine Roullier[1,*]

[1] Nantes Université, Institut des Substances et Organismes de la Mer, ISOMer, UR 2160, F-44000 Nantes, France.
[2] Univ Brest, INRAE, Laboratoire Universitaire de Biodiversité et Écologie Microbienne, F-29280 Plouzané, France
* Correspondence: Catherine.roullier@univ-nantes.fr; Tel.: +33251125686

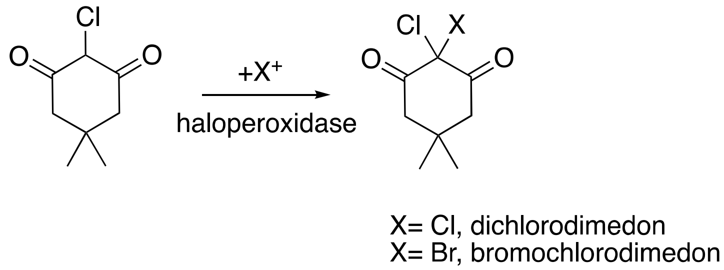

Figure S1. MCD assay: monochlorodimedon is halogenated to obtain dichlorodimedone if $X^+=Cl^+$ or bromochlorodimedon if $X^+= Br^+$.

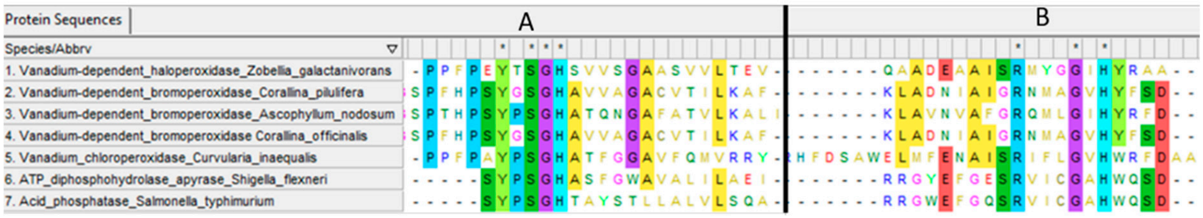

Figure S2. Alignment between vHPOs (fungi, algae, and bacteria) and bacterial acid phosphatases PAP2.

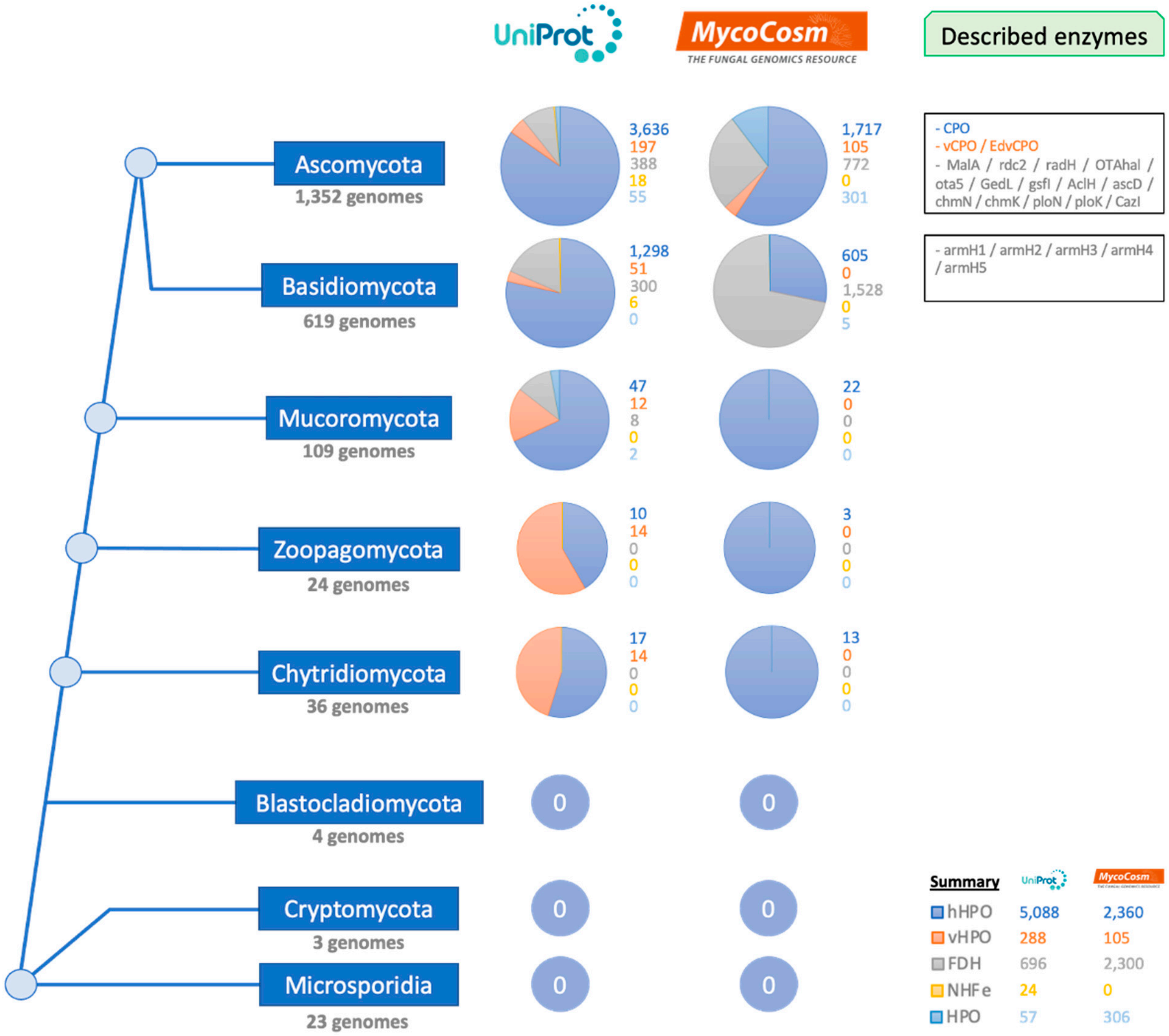

Figure S3. Summary of all putative sequences of fungal halogenation enzymes detected in both UniProt and MycoCosm databases. Distribution of all the detected sequences is found in each pie chart. The number of genomes for each phylogenetic branch available on MycoCosm database is given in the grey under each fungal division. The number of sequences found in each database in January 2022 is indicated next to the caption in the summary part. All described enzymes are presented in the rectangle boxes in front of the corresponding fungal division.
